# Role of IQGAP1 in Papillomavirus-Associated Head and Neck Tumorigenesis

**DOI:** 10.3390/cancers13092276

**Published:** 2021-05-10

**Authors:** Tao Wei, Suyong Choi, Darya Buehler, Denis Lee, Ella Ward-Shaw, Richard A. Anderson, Paul F. Lambert

**Affiliations:** 1 McArdle Laboratory for Cancer Research, School of Medicine and Public Health, University of Wisconsin, Madison, WI 53705, USA; twei27@wisc.edu (T.W.); denis.lee@wisc.edu (D.L.); etward@wisc.edu (E.W.-S.); 2 School of Medicine and Public Health, University of Wisconsin, Madison, WI 53705, USA; schoi58@wisc.edu (S.C.); raanders@wisc.edu (R.A.A.); 3 Department of Pathology and Laboratory Medicine, School of Medicine and Public Health, University of Wisconsin, Madison, WI 53705, USA; buehler2@wisc.edu

**Keywords:** papillomavirus, HPV, head and neck cancer, IQGAP1, PI3K signaling, MmuPV1, infection model, mouse model

## Abstract

**Simple Summary:**

Human papillomaviruses (HPVs) are the most common sexually transmitted pathogens in the United States and are associated with 25% of head and neck cancers (HNCs). To study the genesis of papillomavirus-associated HNC in a physiologically relevant pre-clinical model, we recently developed an infection-based murine model that uses the recently discovered mouse papillomavirus, MmuPV1. In this MmuPV1 HNC model, as in HPV-associated HNCs, the PI3K/AKT/mTOR signaling pathway is upregulated. Components of this pathway are known to be assembled by the scaffolding protein IQGAP1. Utilizing our MmuPV1 HNC model, we tested and demonstrated the importance of IQGAP1 in papillomavirus-induced HNC, in which IQGAP1 is required for optimal induction of the PI3K/AKT/mTOR pathway by MmuPV1 and contributes quantitatively to MmuPV1-induced HNC. Further investigation into how IQGAP1 promotes disease progression may shed additional insights into papillomavirus-induced carcinogenesis and provide new drug targets for treating HPV-associated neoplastic disease.

**Abstract:**

Approximately 25% of head and neck squamous cell carcinomas (HNSCC) are associated with human papillomavirus (HPV) infection. In these cancers as well as in HPV-associated anogenital cancers, PI3K signaling is highly activated. We previously showed that IQ motif-containing GTPase activating protein 1 (IQGAP1), a PI3K pathway scaffolding protein, is overexpressed in and contributes to HNSCC and that blocking IQGAP1-mediated PI3K signaling reduces HPV-positive HNSCC cell survival and migration. In this study, we tested whether IQGAP1 promotes papillomavirus (PV)-associated HNSCCs. IQGAP1 was necessary for optimal PI3K signaling induced by HPV16 oncoproteins in transgenic mice and MmuPV1 infection, a mouse papillomavirus that causes HNSCC in mice. Furthermore, we found that, at 6 months post-infection, MmuPV1-infected *Iqgap1^−/−^* mice developed significantly less severe tumor phenotypes than MmuPV1-infected *Iqgap1^+/+^* mice, indicating a role of IQGAP1 in MmuPV1-associated HNSCC. The tumors resulting from MmuPV1 infection showed features consistent with HPV infection and HPV-associated cancer. However, such IQGAP1-dependent effects on disease severity were not observed in an HPV16 transgenic mouse model for HNC. This may reflect that IQGAP1 plays a role in earlier stages of viral pathogenesis, or other activities of HPV16 oncogenes are more dominant in driving carcinogenesis than their influence on PI3K signaling.

## 1. Introduction

Papillomaviruses (PVs) are species-specific, non-enveloped, double-stranded small (~8 kbp) DNA viruses. High-risk human papillomaviruses (HPVs), such as HPV16, 18, and 33, cause 5% of all human cancers [1,2], including 25% of head and neck squamous cell carcinomas (HNSCCs) and, in particular, those arising in the oropharynx [1,3]. With the prevalence of cigarette smoking, another major risk factor for HNSCC, declining in the U.S., the percentage of HNSCCs associated with HPVs has increased over the past several decades [4,5,6,7], emphasizing the importance of studying HPV-positive HNSCC. HNSCC is the sixth most common cancer worldwide [8]. Because of the poor uptake of the HPV vaccine in the U.S. and its limited availability in many countries worldwide [9,10], HPV-associated cancers, including HNSCC, will remain common cancers for the foreseeable future and are therefore worthy of further study.

PI3K signaling is highly implicated in HPV-associated cancers [11,12,13,14,15,16,17]. *PIK3CA*, the gene that encodes for the catalytic subunit of PI3K, is altered in 56% of HNSCCs [11]. Most of these alterations are activating *PIK3CA* mutations, which result in upregulated PI3K signaling that can promote HNSCC cell growth, tumor progression, invasion, and metastasis [18,19,20]. Recently, IQ motif-containing GTPase-activating protein 1 (IQGAP1) was reported to act as a scaffold for PI3K signaling [21]. IQGAP1 is overexpressed in many human cancers, including HNSCCs, and regulates multiple cellular activities, including but not limited to cytoskeletal dynamics, cell proliferation, cell–cell adhesion, and invasion [22,23,24]. Importantly, high levels of IQGAP1 also correlate with worse prognosis in HNSCC patients [25,26]. In our previous study, IQGAP1 contributed quantitatively to HNSCC in the absence of HPV, at least partly through PI3K signaling [26]. However, there are several lines of evidence that IQGAP1 may also be important in HPV-positive HNSCC. For instance, IQGAP1-mediated PI3K signaling contributes to HPV-positive HNSCC survival and migration [26,27]. In another study, PI3K signaling was also upregulated in PV-induced head and neck tumors [28]. Given the importance of PI3K in HPV-associated cancers, we hypothesized that IQGAP1 also plays a role in PV-associated head and neck tumorigenesis.

Animal models are extremely valuable for studying the contributions of viral oncoproteins and host factors in PV-associated HNSCCs [29]. In *K14-HPV16E6/E7* (*K14E6E7*) transgenic mice, expression of the HPV16 viral oncogenes, E6 and E7, is targeted to the basal layer of the stratified epithelium [29,30,31]. Paired with the synthetic oral carcinogen 4-Nitroquinoline 1-oxide (4NQO) [32], *K14E6E7* mice develop poorly differentiated, high-grade, invasive keratinizing squamous cell carcinomas [33]. In another HPV transgenic model, a doxycycline-inducible mouse model, in which the HPV16 E6 and E7 oncoproteins are expressed, DMBA and TPA carcinogen treatment promoted HNSCC development [17]. Research is now expanded to infection-based models with the discovery of mouse papillomavirus (MmuPV1) [34]. MmuPV1 can infect and cause cancer in laboratory mice in both cutaneous and mucosal surface epithelia [28,35,36,37,38], providing new opportunities to study multiple aspects of PV-associated tumorigenesis from the point of infection. We previously established an infection-based model for studying PV-associated HNSCC using MmuPV1 [28]. Together with the cofactors 4NQO and ultraviolet radiation B (UVB), MmuPV1 induced invasive SCC in immunocompetent mice. The resulting tumors contained both HPV infection and HPV-associated cancer features, including increased levels of PI3K signaling [28], indicating the possible role of PI3K signaling in the MmuPV1-induced pathogenesis and associated tumorigenesis. Therefore, we decided to utilize this newly established model to study the role of IQGAP1 in PV-associated HNSCC.

In this study, we demonstrated that IQGAP1 was necessary for efficient HPV oncoprotein-induced PI3K signaling. We also found that PI3K signaling was highly induced in oral keratinocytes upon MmuPV1 infection, which was attenuated in IQGAP1-deficient cells. In an in vivo tumorigenesis study, MmuPV1-infected *Iqgap1^+/+^* mice developed significantly more severe tumor phenotypes at six months post-infection than MmuPV1-infected *Iqgap1^−/−^* mice. MmuPV1-induced tumors showed features commonly found in HPV-infected human tissues and HPV-associated cancer, consistent with our previously published data. We also tested the role of IQGAP1 using the *K14E6E7* transgenic mouse model. Here, we did not observe a significant effect of IQGAP1 on head and neck tumorigenesis. This result may reflect that IQGAP1 is involved in earlier stages of viral-induced neoplastic progression, or HPV16 oncogenes drive carcinogenesis primarily through their other, well-known effects on the cellular tumor suppressors, p53 and pRb.

## 2. Methods and Materials

### 2.1. Cell Culture

**Figure 1:**
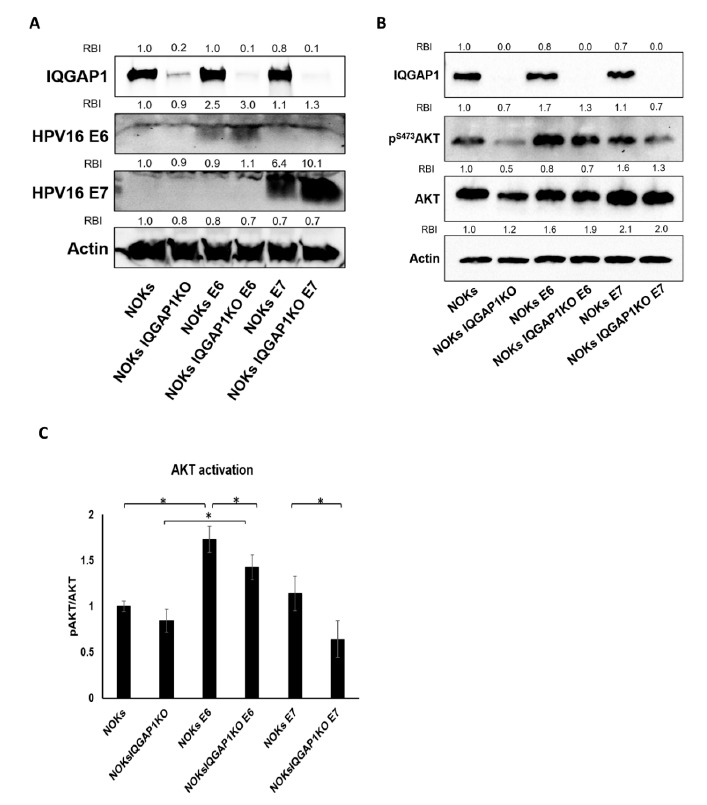
IQGAP1 is necessary for efficient HPV oncoprotein-induced PI3K signaling. (**A**) Immunoblot to confirm the expression of IQGAP1, HPV16 E6, and HPV16 E7 in the constructed IQGAP1^KO^ NOKs-HPV cell lines. Relative band intensity (RBI) was calculated by normalizing to the band intensity of NOKs lane. (**B**) Immunoblot shows that stable expression of HPV16 E6 and E7 regulates PI3K signaling in NOKs cell, in part depending on IQGAP1. RBI was calculated by normalizing to NOKs lane. (**C**) Quantification of immunoblot in (**B**). AKT activation was determined by calculating the ratio of p^S473^AKT over the levels of total AKT. The intensity of the immunoblots was analyzed by ImageJ, and the graph shows mean ± standard deviation of three independent experiments. Statistics: NOKs vs. NOKs IQGAP1^KO^, *p* = 0.12; NOKs vs. NOKs E6, *p* = 0.001; NOKs E6 vs. NOKs IQGAP1^KO^ E6, *p* = 0.05; NOKs IQGAP1^KO^ vs. NOKs IQGAP1^KO^ E6, *p* = 0.005; NOKs vs. NOKs E7, *p* = 0.34; NOKs E7 vs. NOKs IQGAP1^KO^ E7, *p* = 0.03; NOKs IQGAP1^KO^ vs. NOKs IQGAP1^KO^ E7, *p* = 0.19. All statistical analysis in this figure was conducted with two-sided *t*-test. Asterisks represent statistical significance. Note: all uncropped western blot images for this study are summarized in Figure S8.

**Figure 2:**
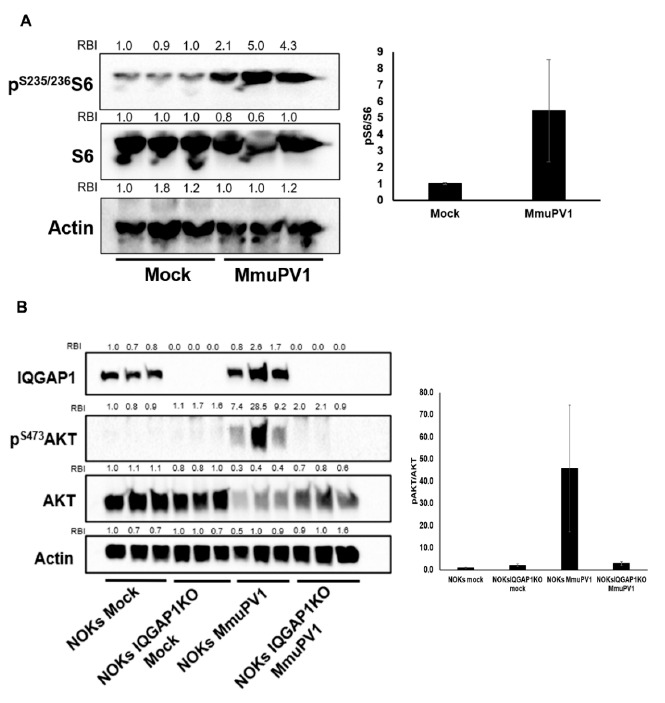
MmuPV1 infection induces PI3K signaling in keratinocytes. (**A**) MmuPV1 infection induces PI3K signaling in primary mouse keratinocytes. Left: immunoblotting detection of S6 activation levels in mock-infected and MmuPV1-infected primary mouse keratinocytes. Relative band intensity (RBI) was calculated by normalizing to the first mock-infected lane from the left. Right: quantification of the band intensity. PI3K signaling level was represented by the ratio of phosphorylated S6 (activated form of S6 protein) over total S6. Statistics: mock vs. MmuPV1, *p* = 0.069, two-sided *t*-test. (**B**) MmuPV1 infection induces PI3K signaling in NOKs via an IQGAP1-dependent mechanism. Left: immunoblot detection of AKT activation levels in mock-infected and MmuPV1-infected NOKs and NOKs IQGAP1^KO^ cells. RBI was calculated by normalizing to the first mock-infected NOKs lane from the left. Right: quantification of band intensity. The PI3K signaling level was represented by the ratio of phosphorylated AKT (activated form of AKT protein) over total AKT. Statistics: NOKs mock vs. NOKs MmuPV1, *p* = 0.053; NOKs MmuPV1 vs. NOKs IQGAP1^KO^ MmuPV1, *p* = 0.06; NOKs IQGAP1KO Mock vs. NOKs IQGAP1KO MmuPV1, *p* = 0.25, two-sided *t*-test. Note: all uncropped western blot images for this study are summarized in Figure S8.

Normal oral keratinocytes (NOKs, used in Figure 1 and Figure 2) were a gift from Dr. Karl Munger (Tufts University School of Medicine, Boston, MA, USA) [39] and were maintained in KSFM supplemented with human epidermal growth factor and bovine pituitary extract (Gibco™, Thermo Fisher Scientific, Inc., Waltham, MA, USA). NOKs E6 and NOKs E7 were generated by transducing pLXSN-16E6 (Addgene #52395, Watertown, MA, USA) and pLXSB-16E7 (cloned from LXSN-E7, Addgene) via retroviruses. Transduced cells were maintained under 300 µg/mL Neomycin (G418, Gibco™, Thermo Fisher Scientific, Inc., Waltham, MA USA) or 7 µg/mL Blasticidin (Gibco™, Thermo Fisher Scientific, Inc.). Mouse keratinocytes (used in Figure 2) were maintained on mitomycin C-treated J2 3T3 feeder cells in complete F medium. Complete F medium consists of 3 parts of F12 and 1 part of DMEM, supplemented with 5% FBS, 24 μg/mL adenine, 8.4 ng/mL cholera toxin, 10 ng/mL epidermal growth factor, 2.4 μg/mL hydrocortisone, 5 μg/mL insulin, and pen/strep.

### 2.2. CRISPR-Cas9 Cell Line Generation

Guide RNAs (gRNAs) to IQGAP1 were designed and cloned into the BsmBI sites of the LentiCRISPRv.2 plasmid (Addgene, #52961): IQGAP1_F: CACCGCCTGTCGAACTAAGTATCCA; IQGAP1_R: AAACTGGATACTTAGTTCGACAGGC. Lentivirus was made and isolated from 293^FT^ cells that were co-transfected with the generated plasmids containing gRNAs and packaging plasmids pCMV-VSV-g (Addgene, #8454) and psPAX2 (Addgene, 12260). NOKS cells were then transduced with purified lentivirus and allowed to undergo selection in the presence of puromycin. Pooled NOKS cells were single cell cloned and screened for loss of IQGAP1 via immunoblotting.

### 2.3. Immunoblotting

Cells were collected as cell pellets and later lysed with RIPA buffer (25mM Tris (pH 8), 150 mM NaCl, 0.1% SDS, 0.5% sodium deoxycholate, 1% Triton X-100) with protease and phosphatase inhibitors. Frozen back skin was cut into small pieces on ice using razor blades, homogenized in 300 μL RIPA using a plastic pestle (Axygen™, Corning Inc., Corning, NY, USA), and incubated on an orbital shaker at 4 °C for 20 min. Homogenates were centrifuged at 14,000 rpm for 20 min at 4 °C, and supernatants (protein lysates) were collected. Protein concentrations were determined using the Bradford Protein Assay (Bio-Rad, Hercules, CA, USA). Equivalent amounts of protein were resolved on precast Mini-PROTEAN TGX 7.5% gels (Bio-Rad) and transferred to PVDF membranes (Millipore, Burlington, MA, USA). Membranes were blocked with 5% nonfat dry milk in TBST (tris-buffered saline with 0.1% Tween-20). Primary antibodies used were summarized in Table S1. Horseradish peroxidase-conjugated secondary antibodies (1:10,000) (Jackson ImmunoResearch, West Grove, PA, USA) and chemiluminescent substrates (Clarity ECL Substrates; Bio-Rad) were used to visualize on a Bio-Rad ChemiDoc Imaging System., version 6.0.1)

### 2.4. Mice

**Figure 3:**
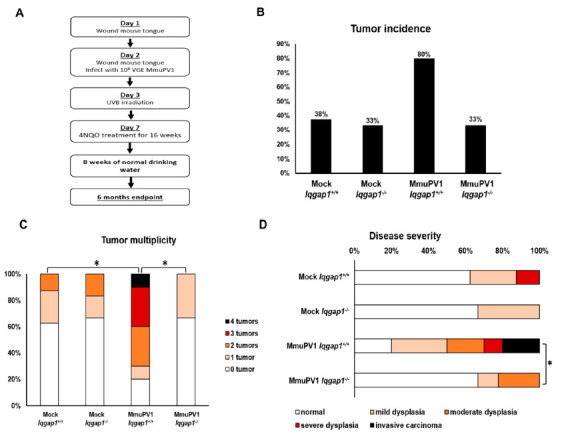
IQGAP1 contributes to MmuPV1-induced head and neck tumorigenesis. (**A**) Overview of experimental protocol for MmuPV1 infection of tongue tissue in *Iqgap1^+/+^* and *Iqgap1^−/−^* mice. (**B**) Tumor incidence in each experimental group at 6 months post-infection. Statistical analysis was performed with the two-sided Fisher’s exact test: MmuPV1-infected *Iqgap1^+/+^* vs. mock-infected *Iqgap1^+/+^*, *p* = 0.14; MmuPV1-infected *Iqgap1^+/+^* vs. MmuPV1-infected *Iqgap1^−/−^*, *p =* 0.069; mock-infected *Iqgap1^+/+^* vs. mock-infected *Iqgap1^−/−^*, *p =* 1; mock-infected *Iqgap1^−/−^* vs. MmuPV1-infected *Iqgap1^−/−^*, *p =* 1, two-sided Fisher’s exact test. (**C**) Tumor multiplicity at 6 months post-MmuPV1 infection. Statistical analysis was performed with the two-sided Wilcoxon rank-sum test: MmuPV1-infected *Iqgap1^+/+^* vs. mock-infected *Iqgap1^+/+^*= 2.1 vs. 0.5, *p =* 0.02; MmuPV1-infected *Iqgap1^+/+^* vs. MmuPV1-infected *Iqgap1^−/−^*= 2.1 vs. 0.3, *p =* 0.006. Mock-infected *Iqgap1^+/+^* vs. mock-infected *Iqgap1^−/−^*, *p =* 1; mock-infected *Iqgap1^−/−^* vs. MmuPV1-infected *Iqgap1^−/−^*, *p =* 0.89. (**D**) Disease severity at 6 months post-infection. Statistical analysis was performed with the two-sided Wilcoxon rank-sum test. MmuPV1-infected *Iqgap1^+/+^* vs. MmuPV1-infected *Iqgap1^−/−^*= 1.8 vs. 0.56, *p =* 0.04; mock-infected *Iqgap1^+/+^* vs. mock-infected *Iqgap1^−/−^*= 0.6 vs. 0.3, *p =* 0.82; MmuPV1-infected *Iqgap1^+/+^* vs. mock-infected *Iqgap1^+/+^*=1.8 vs. 0.6, *p =* 0.06; mock-infected *Iqgap1^−/−^* vs. MmuPV1-infected *Iqgap1^−/−^* = 0.3 vs. 0.56, *p =* 0.84. Asterisks represent statistical significance.

**Figure 4:**
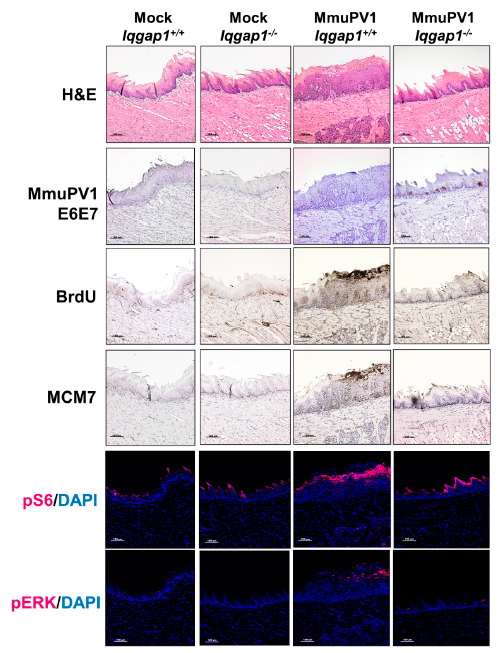
Biomarker analysis of MmuPV1-associated cytoplastic mild dysplastic lesions arising in *Iqgap1^+/+^* and *Iqgap1^−/−^* mice. The presence of MmuPV1 was detected by in situ hybridization against the MmuPV1 E6/E7 transcripts. MCM7 and BrdU were detected by immunohistochemistry. pERK and pS6 were detected by TSA-enhanced immunofluorescence (red: pERK or pS6; blue: DAPI).

*Iqgap1^−/−^* mice [40] (from Dr. David Sacks, National Institutions of Health, Bethesda, MD, USA) were received on a mixed genetic background (*129* and *C57BL/6*) and backcrossed to *FVB* background. At the fifth backcross generation (N5), *Iqgap1^−/−^* mice were crossed with HPV16 transgenic mice (*K14E6E7*, *FVB* background) to generate the experimental groups for studying the role of IQGAP1 in HPV-associated carcinogenesis (Figures S3 and S4, Table S2. All groups were at the same mixed background, sixth backcross generation). The backcrossing of *Iqgap1^−/−^* onto *FVB* continued until reaching N10. Wild-type *FVB* mice (Taconic, Germantown, NY, USA) and *Iqgap1^−/−^* on *FVB* background were used for the mouse papillomavirus (MmuPV1) infection experiment (Figure 3, Figure 4 and Figure S2). All mice were housed in the Association for Assessment of Laboratory Animal Care-approved McArdle Laboratory Animal Care Unit. All procedures were carried out in accordance with an animal protocol approved by the University of Wisconsin Institutional Animal Care and Use Committee.

### 2.5. Primary Mouse Keratinocyte Isolation and Cell Line Establishment

We modified a previously published protocol [41] to isolate primary murine keratinocytes. Briefly, 4-day-old *C57BL/6* pups were sacrificed, and torso skin was allowed to soak in 0.25% trypsin/EDTA overnight at 4 °C. Using forceps and a razor blade, the epidermis was peeled from the dermis and minced. The resulting cells were stirred at 37 °C in normal growth media for one hour, at which point they were passed through a cell strainer and pelleted. The pelleted cells were plated onto mitomycin C-treated 3T3 fibroblasts and cultured in F media supplemented with ROCK inhibitor (Selleck Chemicals S1049, Houston, TX, USA).

### 2.6. MmuPV1 Infection of Keratinocytes

MmuPV1 virus stock was generated by isolating virions from papillomas developed on nude mice as described previously [38]. NOKs and NOKs IQGAP1^KO^ keratinocytes were plated at 2 × 10^5^ cells in 6 cm dishes in KSFM, while the same number of mouse keratinocytes were plated on mitomycin-treated 3T3 feeders in 6 cm plates in complete F media. The next day, feeders were removed by light trypsinization before infection. The keratinocytes were then infected by switching to media containing MmuPV1 at a total of 5 × 10^8^ VGE or the same media containing an equivalent amount of PBS (mock infection). Three hours later, the infected cells were changed to fresh media and incubated at 37 °C for 48 h. Cells were harvested, and protein lysates were prepared to check for signaling levels or total RNA extracted to confirm infection. Total RNA was extracted from infected cells using the RNeasy kit (Qiagen, Hilden, Germany) and reverse-transcribed into cDNA using the QuantiTect Reverse Transcription Kit (Qiagen). E1^E4 transcripts were detected by quantitative PCR (ABI 7900HT) using TaqMan probe [42]. GAPDH (Mm99999915_g1, Thermo Fisher Scientific) was used as a positive control.

### 2.7. MmuPV1 Infection of Tongue Epithelium

The MmuPV1 tongue epithelium infection model was adapted from a previous publication [28]. Briefly, mice were put under isoflurane-induced anesthesia until surgical tolerance stage. Then, the mice were removed from the anesthesia-inducing chamber. Briefly, the tongue was drawn out using flat top forceps and lightly wound on the dorsal surface using an 18-gauge syringe needle. A total of 24 h later, the mice were wound lightly the same way at the same site, and either PBS only (mock infection) or 10^8^ VGE of MmuPV1 were then pipetted onto the wounded site. Over the experimental period, mice were checked monthly for overt tumor formation. At 24 h post-infection, all mice were exposed to a single dose of UVB at 1000 mJ/cm^2^ as described before [38,43]. UVB irradiation was delivered using a custom-designed Research Irradiation Unit (Daavlin, Bryan, OH, USA).

### 2.8. Oral Swabbing and Detection of MmuPV1 E2 by qPCR

Tongues of anesthetized mice were drawn out by forceps, and the infection sites were swabbed multiple times using a flat toothpick. The portion of the toothpick exposed to the tongue was placed into 50 µL of sterile PBS and stored at −80 °C. DNA from the swab samples was isolated using the DNeasy Blood and Tissue kit (Qiagen, #69506, Hilden, Germany) and quantified using Nanodrop to ensure same amount of DNA was loaded for each sample (3 ng). SYBR Green was then used for quantitative PCR with previously published MmuPV1 E2 primers [36].

### 2.9. BrdU Incorporation

We measured bromodeoxyuridine (BrdU) incorporation to evaluate levels of DNA synthesis. Mice were intraperitoneally injected with 250 µL of BrdU (Sigma-Aldrich, St. Louis, MO, USA. 12.5 mg/mL in PBS, stored at −20 °C) one hour before euthanasia. Tissues were harvested and processed for immunohistochemistry.

### 2.10. Immunohistochemistry

Representative slides were selected based on histology scoring, and at least three samples were included for each group. Tissue sections were deparaffinized in xylenes and rehydrated in graded ethanol. Heat-induced antigen unmasking was performed in 10 mM citrate buffer (pH = 6). Slides were treated with 3% H_2_O_2_ in methanol for 10 min at room temperature (RT), washed and blocked with 2.5% horse serum for 1 h at RT, and then incubated in primary antibody at 4 °C overnight in a humidified chamber. M.O.M.^®^ ImmPRESS^®^ HRP polymer kit (Vector laboratories, MP-2400, Burlingame, CA, USA) was applied the next day for 1 h at RT for secondary antibody incubation. Slides were then incubated with 3,3′-diaminobenzidine (Vector Laboratories) and counterstained with hematoxylin. All images were taken with a Zeiss AxioImager M2 microscope using AxioVision software version 4.8.2. (Carl Zeiss Microscopy, LLC., White Plains, NY, USA)

For immunofluorescence, both pS6 and pERK signals were detected using a tyramide-based signal amplification (TSA) method [44]. A detail protocol for TSA was published: https://www.protocols.io/view/untitled-protocol-i8cchsw, (accessed date 8 May 2021). Tissue sections were deparaffinized and subjected to antigen unmasking and H_2_O_2_ treatment as described above, blocked in TSA blocking buffer (Perkin Elmer #FP1012, Waltham, MA, USA). After overnight incubation in primary antibody, slides were incubated in anti-rabbit hrp (1:500) for 1 h at RT and proceeded for TSA treatment (1:500 biotin-tyramide in working reagent). Indicated slides were then probed with K14 antibody. For fluorescent secondary antibody, slides were probed with Alexa-anti-rabbit-488 or SA-647 at 1:200 for 1 h at RT in the dark. Sections were counterstained with DAPI and mounted in Prolong Diamond Antifade Mountant (Invitrogen, Thermo Fisher Scientific Inc.). Information for all primary antibodies is summarized in Table S1.

### 2.11. RNA In Situ Hybridization

Representative slides were selected for each MmuPV1-infected mouse sample based on histology scoring along with 2 representative slides from each corresponding mock-infected group. In situ hybridization was performed using RNAscope 2.5 HD Assay-Brown (Advanced Cell Diagnostic, Newark, CA, USA) according to the manufacturer’s instructions [45]. Signals for the viral transcript were detected using MmuPV1 E6/E7 probes (#409771, Advanced Cell Diagnostic).

### 2.12. 4-Nitroquinoline-1-Oxide (4NQO) Induced Head and Neck Carcinogenesis Study

Adult *Iqgap1^+/+^*, *Iqgap1^−/−^*, *Iqgap1^+/+^K14E6E7*, *Iqgap1^−/−^K14E6E7* mice (between 6 and 8 weeks of age) were treated with the carcinogen 4NQO (Sigma-Aldrich, St. Louis, MO, USA) in their drinking water at a concentration of 10 μg/mL (stored at 4 °C as a 1 mg/mL in propylene glycerol (VWR, Radnor, PA, USA), dilute with water upon use) for 16 weeks. The mice were then returned to regular drinking water for 8 weeks.

### 2.13. Overt Tumor and Histological Analyses

At the endpoint, mice were euthanized, and the numbers of grossly visible, overt tumors on the tongue were quantified. The tissues were then collected, fixed in 4% paraformaldehyde for 24 h followed by 70% ethanol for 24 h, processed, paraffin-embedded, and sectioned at 5 micron intervals. Every tenth section was stained with H&E and examined by a pathologist, DB, in a blinded fashion to assess the presence and the severity of squamous dysplasia (mild, moderate, severe/CIS) and invasive carcinoma, which could be graded as well-differentiated (grade 1), moderately-differentiated (grade 2), poorly-differentiated (grade 3), or sarcomatoid (grade 4).

### 2.14. Statistical Analysis

For disease severity, each microscopic tumor grade was assigned a ranking order (dysplasia: mild = 1, moderate = 2, severe = 3; invasive carcinoma: grade 1 = 4, grade 2 = 5, grade 3 = 6, grade 4 = 8). Wilcoxon rank-sum test was performed to determine the significance of differences in disease severity using MSTAT statistical software version 6.4.2 (https://oncology.wisc.edu/mstat/, accessed on 8 May 2021). Fisher’s exact test was performed to determine the significance of differences in infection incidence between experimental groups at the endpoint using MSTAT.

## 3. Results

### 3.1. IQGAP1 Is Necessary for HPV-Induced PI3K Signaling

Multiple reports described relationships between high-risk HPV oncoproteins, E6 and E7, and PI3K signaling, but the findings varied [46,47,48]. Therefore, we first examined whether HPV16 E6 and E7 regulate PI3K signaling in human keratinocytes, the natural host cells for papillomavirus infection. We used lentiviral transduction to introduce HPV16 E6 and E7 into normal oral keratinocytes (NOKs), which are hTert-immortalized human gingival epithelial cells [39]. To study the role of IQGAP1, the *IQGAP1* gene was knocked out (KO) in these cells using the CRISPR-Cas9 system. The status of IQGAP1 and HPV protein expression in these cells was confirmed by immunoblotting (Figure 1A). Utilizing these cells, we found that E6 expression significantly increased levels of pAKT, and this increase was significantly reduced in the IQGAP1 KO cells (Figure 1B,C; NOKs vs. NOKs E6, *p* = 0.001; NOKs E6 vs. NOKs IQGAP1^KO^ E6, *p* = 0.05, two-sided *t*-test). On the contrary, E7 expression did not significantly induce PI3K signaling, but the absence of IQGAP1 still reduced PI3K signaling in the E7-expressing cells (Figure 1B,C; NOKs vs. NOKs E7, *p* = 0.34; NOKs E7 vs. NOKs IQGAP1^KO^ E7, *p* = 0.03, two-sided *t*-test). Together, these data support the hypothesis that the HPV16 oncoproteins can increase PI3K signaling in human oral keratinocytes, in which E6 appears to have a stronger effect than E7 in inducing the signaling. In both cases, this upregulation depends on the IQGAP1 protein.

### 3.2. MmuPV1 Infection Upregulates PI3K Signaling in Keratinocytes

The recent discovery of the mouse papillomavirus (MmuPV1), which infects laboratory mice and causes cancer in both cutaneous and mucosal epithelia, provides new opportunities to study papillomavirus infection and disease progression in a tractable and genetically modifiable host [34,35,36,37,38]. We previously established an infection-based model for PV-associated HNSCC using MmuPV1 [28]. Interestingly, PI3K signaling was upregulated in these MmuPV1-induced tumors [28], as is observed in HPV-associated cancers [12,13]. Because IQGAP1 is important for HPV-induced PI3K signaling (Figure 1), we decided to test the hypothesis that IQGAP1 also mediated MmuPV1-induced PI3K signaling.

We first explored the potential role of IQGAP1 in mediating MmuPV1-induced PI3K mediated signaling by infecting murine keratinocytes in vitro. We isolated primary mouse keratinocytes from neonatal skin and confirmed that these cells were permissive to MmuPV1 infection by measuring the accumulation of MmuPV1 E1^E4 spliced transcripts (Figure S1A). The E1^E4 spliced event is common to most mature mRNAs arising in MmuPV1-infected cells [45]. We then infected these primary mouse keratinocytes and, 48 h later, harvested protein and measured phospho-S6 (pS6) levels, a downstream protein in PI3K signaling cascade. We chose pS6 because we had greater success detecting mouse pS6 than mouse pAKT. We measured higher levels of pS6 in the MmuPV1-infected primary mouse keratinocytes compared to mock-infected controls (Figure 2A), suggesting a possible role of PI3K signaling in MmuPV1 infection and life cycle.

In order to investigate whether MmuPV1-induced PI3K signaling is regulated by IQGAP1, we again turned to our isogenic NOKs and NOKs IQGAP1^KO^ cells based upon the prior observation that MmuPV1 can infect human keratinocytes [49]. We first confirmed that human NOKs and NOKs IQGAP1^KO^ cells could be infected by MmuPV1 (Figure S1B). Both NOKs and NOKs IQGAP1^KO^ produced similar levels of E1^E4 spliced transcripts upon MmuPV1 infection, indicating that MmuPV1 can also infect these human keratinocytes. We then infected the NOKs and the NOKs IQGAP1^KO^ cells using the same amount of virus and quantified the levels of pAKT by immunoblotting at 48 h post-infection. MmuPV1 infection triggered PI3K signaling strongly in NOKs cells but failed to do so in NOKs IQGAP1^KO^ cells (Figure 2B). This indicates that MmuPV1 can induce PI3K signaling upon infection in an IQGAP1-dependent manner. We also observed a downregulation of total AKT levels upon MmuPV1 infections in both NOKs and NOKs IQGAP1^KO^ cells, the reason for which needs further exploration. Together, these data in human and mouse keratinocytes confirm that MmuPV1 induces PI3K signaling and that this signaling may depend on IQGAP1.

### 3.3. IQGAP1 Contributes to PV-Associated Head and Neck Tumorigenesis in an Infection-Based Model

With the knowledge that MmuPV1 induces PI3K signaling in an IQGAP1-dependent mechanism in vitro, we next investigated the role of IQGAP1 in PV-associated head and neck tumorigenesis in vivo using the MmuPV1 infection model we previously established [28]. The experimental design is summarized in Figure 3A. Briefly, we wound the tongues of wild-type *FVB* mice (*Iqgap1^+/+^*) and the *Iqgap1-knockout* mice on the *FVB* background (*Iqgap1^−/−^*). The next day (day 2), the tongues were wound again and infected with either PBS (mock-infected) or 10^8^ VGE of MmuPV1 (MmuPV1-infected), the same dosage found to cause tumors in our published model. All mice were treated with 1000 mJ of UVB radiation on day 3 to induce immunosuppression [38]. Starting at day 7, mice were given drinking water containing 20 µg/mL of the synthetic oral carcinogen, 4-nitroquinoline-1-oxide (4NQO) for 16 weeks, followed by 8 weeks of regular drinking water (no carcinogen). At the 6 month endpoint, mice were sacrificed, and their tongues were collected, fixed, processed, sectioned at 5 micrometer intervals, and every tenth slide was stained with H&E. The histology of the resulting tissues was assessed using the H&E-stained slides, and the disease state (normal, dysplasia, and invasive carcinoma) was scored by a trained pathologist.

To confirm the state of MmuPV1 infection in the infected sites within the oral cavity of mice, we collected oral swab samples from all experimental mice at 3 weeks post-infection. DNA was extracted from these samples, and qPCR was performed using primers specific to the MmuPV1 E2 gene. Samples from MmuPV1-infected mice showed signals for E2 to various degrees, while no samples from mock-infected mice gave any signals above the background level, indicating that viral infection was successfully established in MmuPV1-infected groups but not the mock-infected groups (Figure S2). No significant differences in E2 levels were detected between MmuPV1-infected *Iqgap1^+/+^* and *Iqgap1^−/−^* groups (*p* = 0.86, two-sided *t*-test), suggesting that the status of IQGAP1 did not affect early viral establishment upon infection.

At the 6 month endpoint, we found that IQGAP1 significantly affected several aspects of PV-induced tumorigenesis in the head and the neck (Figure 3B–D). As expected, wild type (*Iqgap1^+/+^*) mice infected with MmuPV1 showed increased tumor incidence of tumors compared to the mock-infected counterparts (Figure 3B, 80% vs. 38%), though this difference was not statistically significant (MmuPV1-infected *Iqgap1^+/+^* vs. mock-infected *Iqgap1^+/+^*, *p* = 0.14; two-sided Fisher’s exact test). MmuPV1-infected *Iqgap1^−/−^* mice also had much lower tumor incidence than the MmuPV1-infected *Iqgap1^+/+^* mice (33% vs. 80%), though this difference also did not quite reach the 95% confidence limit (Figure 3B. MmuPV1-infected *Iqgap1^+/+^* vs. MmuPV1-infected *Iqgap1^−/−^*, *p* = 0.069, two-sided Fisher’s exact test). However, when we compared tumor multiplicity, MmuPV1-infected *Iqgap1^+/+^* mice developed a significantly higher number of tumors per mouse than in mock-infected *Iqgap1^+/+^* mice. MmuPV1-infected *Iqgap1^−/−^* mice showed significantly reduced number of tumors per mouse than MmuPV1-infected *Iqgap1^+/+^* mice to a level comparable to mock-infected mice (Figure 3C; MmuPV1-infected *Iqgap1^+/+^* vs. mock-infected *Iqgap1^+/+^* = 2.1 vs. 0.5, *p* = 0.02; MmuPV1-infected *Iqgap1^+/+^* vs. MmuPV1-infected *Iqgap1^−/−^* = 2.1 vs. 0.3, *p*=0.006; MmuPV1-infected *Iqgap1^−/−^* vs. Mock-infected *Iqgap1^−/−^* = 0.3 vs. 0.5, *p* = 0.89, two-sided Wilcoxon rank-sum test). The analysis of disease severity showed a similar result: MmuPV1-infected *Iqgap1^+/+^* mice developed significantly worse disease by developing higher-grade disease than the MmuPV1-infected *Iqgap1^−/−^* mice (Figure 3D and Table 1. MmuPV1-infected *Iqgap1^+/+^* vs. MmuPV1-infected *Iqgap1^−/−^* = 1.8 vs. 0.56, *p* = 0.04, two-sided Wilcoxon rank-sum test). Together, these results demonstrate that IQGAP1 contributes quantitatively to MmuPV1-induced tumorigenesis, particularly tumor multiplicity and disease severity.

### 3.4. Biomarker Analysis of MmuPV1-Induced Oral Tumors Arising in Iqgap1^+/+^ and Iqgap1^−/−^ Mice

We assessed the MmuPV1-induced tumors with a panel of relevant biomarkers (Figure 4). To confirm the presence of the virus at 6 months post-infection, we used in situ hybridization and detected MmuPV1 E6E7 transcripts in tissues from both MmuPV1-infected groups but not in the mock-infected groups. Consistent with our previous results [28], signals of MmuPV1 transcripts were detected in disease-free epithelial tissues containing pathology within normal limits, which we term subclinical infection sites, as well as within tumors. Several tumors arising from MmuPV1-infected *Iqgap1^+/+^* mice were positive for the presence of the virus. However, only one out of the three tumors, a mild dysplastic lesion, from the MmuPV1-infected *Iqgap1^−/−^* mice was virus-positive. This small sample size in the infected *Iqgap1^−/−^* group hindered our ability to quantitatively compare the molecular differences between MmuPV1-induced tumors arising in *Iqgap1^+/+^* and *Iqgap1^−/−^* animals.

Despite this limitation, we chose to do an analysis comparing one mildly dysplastic lesion from each infected group to look at the expression patterns of two biomarkers associated with HPV infection and related neoplastic disease: bromodeoxyuridine (BrdU) and minichromosome maintenance complex component 7 (MCM7). The incorporation of BrdU, a nucleotide analog, measures the level of DNA synthesis, which is often upregulated in HPV-induced lesions [33]. Increased numbers of BrdU-positive cells could be observed in both infected groups compared to their mock-infected counterparts, indicating a higher level of DNA synthesis in these lesions. MCM7 is an E2F-responsive protein and is commonly upregulated in HPV-infected tissue and epithelia of HPV16 transgenic mice due to the inactivation of retinoblastoma protein (Rb) by the viral protein E7 [33]. MCM7 levels were highly upregulated in the epithelium of MmuPV1-infected *Iqgap1^+/+^* mice, consistent with our previously published data [28,36], showing an increased level of E2F transcription in MmuPV1-infected lesions. The MCM7 level in the lesion from MmuPV1-infected *Iqgap1^−/−^* seemed to be slightly higher than the one in the mock-infected *Iqgap1^−/−^* but much lower than the one from MmuPV1-infected *Iqgap1^+/+^*. However, without additional samples for proper quantitative analysis, it is difficult to conclude whether there were any significant differences in MCM7 expression based on IQGAP1 status.

We also compared two biomarkers related to HPV-associated carcinogenesis. pS6, the active form of the effector S6 for PI3K/mTOR signaling, and phosho-ERK (pERK), the active form of the effector ERK for mitogen-activated protein kinase (MAPK) signaling, are upregulated in HPV-associated cancers according to previous reports [12,13,50,51]. In our study, both pS6 and pERK levels increased in lesions from both infected groups compared to those from mock-infected groups, indicating increased PI3K and MAPK signaling in MmuPV1-induced lesions. The infected-*Iqgap1^−/−^* mice may have qualitatively lower levels of pS6 and pERK compared to the infected-*Iqgap1^+/+^* mice, but further studies are needed to repeat this experiment on a larger scale for quantitative analysis.

### 3.5. IQGAP1 Does Not Impact Head and Neck Carcinogenesis in an HPV16-Transgenic Mouse Model

We also tested the role of IQGAP1 in the *K14E6E7* HPV transgenic mouse model, another commonly used in vivo model to study HPV-associated carcinogenesis [10,14,15,16,17]. We started by checking whether IQGAP1 affects PI3K signaling in the *K14E6E7* mice. We crossed *Iqgap1^−/−^* mice [40] with HPV16 transgenic mice that target the expression of the HPV oncoproteins to stratified epithelium using the K14-promoter (*K14E6E7*, [30,31]). In doing so, we generated four groups of mice: *Iqgap1^+/+^*, *Iqgap1^−/−^*, *Iqgap1^+/+^K14E6E7*, and *Iqgap1^−/−^K14E6E7*. Immunoblot showed that the *Iqgap1^+/+^K14E6E7* mice have higher levels of PI3K signaling than the *Iqgap1^+/+^* mice, with a difference approaching statistical significance (Figure S3, *Iqgap1^+/+^* vs. *Iqgap1^+/+^K14E6E7*, *p =* 0.07, two-sided *t*-test). The loss of IQGAP1 in *Iqgap1^−/−^K14E6E7* mice significantly reduced the PI3K signaling back to a level comparable to the non-HPV groups (Figure S3, *Iqgap1^+/+^K14E6E7* vs. *Iqgap1^−/−^K14E6E7*, *p =* 0.024; *Iqgap1^−/−^* vs. *Iqgap1^−/−^K14E6E7*, *p =* 0.2, two-sided *t*-test). These results indicate that the HPV16 oncoproteins upregulate PI3K signaling in vivo in an IQGAP1-mediated manner.

Since PI3K signaling is highly implicated in HPV-associated HNSCC [11,15], with the knowledge that IQGAP1 is important for the HPV-associated PI3K signaling (Figure S3), we then asked whether IQGAP1 contributes to HPV16-associated carcinogenesis. We utilized a pre-clinical model previously developed in our lab for HPV-associated HNSCC, which induces HNSCC in HPV16 transgenic mice using the synthetic oral carcinogen 4NQO [32,33]. At the age of 6–8 weeks, the four groups of mice generated in Figure S3 started receiving drinking water containing 10 μg/mL 4NQO for 16 weeks, followed by 8 weeks of regular drinking water (no 4NQO) to allow cancers to develop (Figure S4A). At the endpoint of 24 weeks, we sacrificed the mice, counted overt tumors, and collected tissue for histological analysis.

Mice expressing HPV16 E6 and E7 showed significantly higher overt tumor incidence (Figure S4B. *Iqgap1^+/+^* vs. *Iqgap1^+/+^K14E6E7*, *p* < 0.0001; *Iqgap1^−/−^* vs. *Iqgap1^−/−^K14E6E7*, *p =* 0.01, two-sided Fisher’s exact test), consistent with previously published data [33]. However, the loss of IQGAP1 did not significantly impact the tumor incidence (Figure S4B, *Iqgap1^+/+^* vs. *Iqgap1^−/−^*, *p =* 0.7; *Iqgap1^+/+^K14E6E7* vs. *Iqgap1^−/−^K14E6E7*, *p =* 0.6, two-sided Fisher’s exact test). The expression of HPV oncoproteins also significantly increased tumor multiplicity (Figure S4C. *Iqgap1^+/+^* vs. *Iqgap1^+/+^K14E6E7* = 0.9 vs. 3.9, *p* < 0.0001; *Iqgap1^−/−^* vs. *Iqgap1^−/−^K14E6E7 =* 0.6 vs. 3.2, *p* < 0.0001, two-sided Wilcoxon rank-sum test), consistent with our prior studies. However, the loss of IQGAP1 only modestly decreased tumor multiplicity (Figure S4C. *Iqgap1^+/+^* vs. *Iqgap1^−/−^*= 0.9 vs. 0.6, *p =* 0.2; *Iqgap1^+/+^K14E6E7* vs. *Iqgap1^−/−^K14E6E7* = 3.9 vs. 3.2, *p =* 0.9, two-sided Wilcoxon rank-sum test). There was also no significant difference between tumor formation at various mucosal sites (tongue and esophagus) in the presence or the absence of IQGAP1. These results indicate that IQGAP1 does not significantly contribute to HPV-associated tumorigenesis in this model.

In terms of histopathologic examination, HPV16 transgenic mice developed significantly higher incidences of invasive squamous cell carcinoma than non-transgenic groups (Figure S4D, *Iqgap1^+/+^* vs. *Iqgap1^+/+^K14E6E7*, *p* < 0.0001; *Iqgap1^−/−^* vs. *Iqgap1^−/−^K14E6E7*, *p* < 0.0001, two-sided Fisher’s exact test). However, the loss of IQGAP1 did not cause a significant reduction in the incidence of invasive carcinoma (*Iqgap1^+/+^* vs. *Iqgap1^−/−^*, *p =* 1; *Iqgap1^+/+^K14E6E7* vs. *Iqgap1^−/−^K14E6E7*, *p =* 0.26, two-sided Fisher’s exact test). In comparison to *Iqgap1^+/+^K14E6E7* mice, *Iqgap1^−/−^K14E6E7* mice showed a modest decrease in cancer foci multiplicity, but this did not reach statistical significance (Figure S4E. *Iqgap1^+/+^K14E6E7* vs. *Iqgap1^−/−^K14E6E7*= 1.7 vs. 1.3, *p =* 0.26, two-sided Wilcoxon rank-sum test). However, loss of IQGAP1 significantly reduced the number of HPV-associated carcinoma foci, specifically in the esophagus, relative to the tongue (Figures S4F and S5A,B).

The disease severity in mice from all experimental groups is summarized in Table S2 and Figure S5C,D. Similar to previous results, mice expressing the HPV16 oncoproteins showed much more severe disease than their non-HPV transgenic counterparts (Table S2. *Iqgap1^+/+^* vs. *Iqgap1^+/+^K14E6E7*, *p* < 0.0001; *Iqgap1^−/−^* vs. *Iqgap1^−/−^K14E6E7*, *p* < 0.0001, two-sided Wilcoxon rank-sum test). However, the absence of IQGAP1 again did not significantly reduce disease severity (Table S2. *Iqgap1^+/+^* vs. *Iqgap1^−/−^*, *p =* 0.4; *Iqgap1^+/+^K14E6E7* vs. *Iqgap1^−/−^K14E6E7*, *p =* 0.27). Altogether, our results show that, while IQGAP1 is necessary for efficient HPV-induced PI3K signaling (Figure 1 and Figure S3) in the HPV-transgenic mouse model, IQGAP1 did not significantly contribute to HPV-associated HNSCC.

### 3.6. Iqgap1^+/+^K14E6E7 and Iqgap1^−/−^ K14E6E7 Mice Showed Similar Biomarker Patterns

To better understand why IQGAP1 did not impact HPV-associated HNSCC in the *K14E6E7* model, we assessed the mouse tissues with a panel of biomarkers. We first looked at the two markers of HPV infection, MCM7 and BrdU, in the normal epithelia of our experimental mice (Figure S6). As expected, *Iqgap1^+/+^K14E6E7* mice showed upregulated levels of MCM7 and BrdU compared to *Iqgap1^+/+^* mice (Figure S6), indicating that E6 and E7 expression increased E2F transcription and DNA synthesis in these mice. However, *Iqgap1^−/−^K14E6E7* mice did not show reduced levels of either markers than *Iqgap1^+/+^K14E6E7* mice, suggesting that IQGAP1 did not affect HPV-induced E2F transcription and DNA synthesis.

Since IQGAP1 can scaffold for both PI3K and MAPK signaling, we hypothesized that there might be a difference in these pathways within the tumors depending on IQGAP1 status. We tested this hypothesis by assessing pS6 and pERK expression, two markers related to HPV-associated carcinogenesis. In mice expressing E6 and E7, we observed high levels of pS6 and pERK in tumors compared to the adjacent normal epithelia (Figure S7). However, the expressions of neither pS6 nor pERK were consistent throughout the tumor. Instead, we observed patches of signal for both markers within one tumor without any apparent correlation between the biomarkers (Figure S7). Similar variable expression patterns for pS6 and pERK could be observed in both *Iqgap1^+/+^K14E6E7 and Iqgap1^−/−^K14E6E7* mice, indicating that the expression of IQGAP1 did not affect the expression of pS6 or pERK within the HPV-induced tumors. Together, our data showed that, although IQGAP1 is necessary for HPV-induced PI3K signaling in normal epithelia, it did not influence markers related to HPV infection or HPV-associated carcinogenesis, consistent with the previous result that IQGAP1 did not reduce HPV-associated HNSCC (Figures S4 and S5). This may indicate that other molecules compensate for the loss of IQGAP1 to support the necessary signaling for HPV-associated carcinogenesis.

## 4. Discussion

Our study investigated the role of IQGAP1 in PV-associated head and neck tumorigenesis. We first showed that IQGAP1 was necessary for HPV16 oncoproteins to efficiently induce PI3K signaling, both in vitro and in vivo. PI3K signaling was also highly activated in keratinocytes upon in vitro infection with a murine papillomavirus, MmuPV1, at least partly through a mechanism dependent on IQGAP1. Therefore, we decided to study the role of IQGAP1 in PV-associated head and neck tumorigenesis using a newly developed MmuPV1-infection based murine model for HNSCC. Neither *Iqgap1^+/+^* nor *Iqgap1^−/−^* mice infected with MmuPV1 displayed a difference in infection incidence at 3 weeks post-infection, indicating that IQGAP1 protein most likely did not affect viral infectivity. At 6 months post-infection, MmuPV1-infected *Iqgap1^+/+^* mice developed significantly higher-grade tumor phenotypes than MmuPV1-infected *Iqgap1^−/−^* mice, suggesting a role of IQGAP1 in MmuPV1-associated tumorigenesis. The tumors in MmuPV1-infected mice exhibited features of both HPV infection and HPV-associated cancer, consistent with our previously published results [28,36].

We also tested the role of IQGAP1 using our laboratory’s well-established HPV16 transgenic mouse model, in which the expression of HPV16 E6 and E7 is targeted to the basal layer of epithelia using the K14 promoter. Contrary to our results from the MmuPV1 infection-based model, we did not observe any significant contribution of IQGAP1 to carcinogenesis in this HPV16 transgenic mouse model. The tumors arising in *Iqgap1^+/+^K14E6E7* and *Iqgap1^−/−^K14E6E7* mice also displayed a very similar biomarker pattern, indicating that the expression of IQGAP1 also did not noticeably impact HPV-associated carcinogenesis at the molecular level. It is possible that the combined strength of the 4NQO carcinogen treatment and the HPV16 oncoproteins masked any anti-tumor effects resulting from the loss of IQGAP1. In our previous HPV-negative HNSCC studies, we observed such a phenomenon, i.e., a high dose of 4NQO masked the tumor-suppressing effect resulting from an absence of IQGAP1 [26]. Another possibility is that other proteins act to maintain PI3K signaling in the absence of IQGAP1 during cancer progression. One such candidate is IQGAP3, another member in the IQGAP family, a homolog to IQGAP1 [52]. IQGAP3 is often overexpressed in cancer and is implicated as an oncogene [53,54,55,56]. Similar to IQGAP1, IQGAP3 can also scaffold proteins for the Ras-MARK pathway and interact with PI3K family protein [55,56,57], supporting the hypothesis that IQGAP3 may compensate for IQGAP1 loss to maintain signaling. Interestingly, transcriptome analysis showed that IQGAP3 is significantly upregulated during lesion progression in HPV-positive cervical cancer tissue [58], further suggesting that IQGAP3 could be important in HPV-associated carcinogenesis.

While we did not observe a significant contribution of IQGAP1 to HPV-associated carcinogenesis using the HPV16 transgenic mouse model, we did demonstrate that IQGAP1 is important for MmuPV1-associated tumor formation using the infection-based model. The exact reasons for this difference warrant further investigation. One explanation could be that the two models undergo different processes of neoplastic disease formation. The HPV16 transgenic mouse model relies on the overexpression of HPV16 E6 and E7 [29]. HPV16 E6 binds to and degrades p53 together with the E3 ligase E6AP; HPV16 E7 inactivates pRb by binding through its LXCXE motif, which upregulates E2F transcription [59]. By inactivating these two major tumor suppressors, HPV16 oncoproteins force the cell to re-enter the cell cycle, promote cell proliferation, and later lead to cancer [59]. However, MmuPV1 acts differently from HPV16. MmuPV1 E6 inhibits both NOTCH and TGF-β signaling, delaying keratinocyte differentiation and promoting proliferation [60]. MmuPV1 E7 does not contain an LXCXE motif and binds to pRb in an LXCXE-independent manner [37]. Hence, it is reasonable to hypothesize that MmuPV1 primarily induces tumorigenesis through mechanisms other than inactivating p53 and pRb, in which PI3K signaling could be playing a more critical role than in HPV16-associated cancer development.

The timing of IQGAP1-mediated signaling in viral infection and tumor formation might also explain the difference between the two models. HPV16 transgenic mouse model represents the later stages of HPV-associated carcinogenesis, in which HPV16 E6 and E7 are overexpressed, likely due to the integration of the virus into the host genome [59]. The MmuPV1 infection-based model captures a series of events, including viral entry, viral maintenance, viral amplification, virus–host interactions such as with the host immune response, in addition to the later stages of tumorigenesis [37]. Therefore, it is possible that IQGAP1 and IQGAP1-mediated PI3K signaling are essential for processes that occur during the earlier stages of PV-associated pathogenesis, which are not recapitulated in the HPV16 transgenic mouse model. HPV16 pseudovirus infection activates PI3K/mTOR pathway in the host cell to inhibit autophagy [61]. This is consistent with our observation with the upregulation of PI3K signaling in MmuPV1-infected keratinocytes, an effect that can very possibly be IQGAP1-dependent (Figure 2). PI3K signaling is also reported to downregulate HPV16 E6 and E7 expression and therefore induce a dormant-like state of the host cell when necessary [62], which might be important at the early stage of infection to help evade immune detection and maintain the viral genome. When entering the productive life cycle, the virus needs to drive cell proliferation in the basal and the parabasal cell layers [59]. The PI3K pathway may play a role in this process, given its ability to promote proliferation, especially for MmuPV1. The exact mechanisms by which this virus induces pathogenesis are still largely unknown.

Another possibility of how IQGAP1 contributes to PV-associated HNSCC is through the Rac1/Cdc42 signaling [63,64,65,66]. Rac1 is frequently over-activated in HNSCC cell lines [63]. The expression of HPV16 E6 activates Cdc42, while HPV18 E6 activates Rac1 [65,66]. Both Rac1 and Cdc42 are targets of IQGAP1 [67,68]. IQGAP1 can bind to and mediate the activation of both Rac1 and Cdc42 and subsequently regulate cell motility and invasion [31,68,69,70]. Therefore, it is possible that IQGAP1 contributes to PV-associated HNSCC through Rac1 and/or Cdc42. Interestingly, Rac1 is essential for HPV8-associated skin tumorigenesis [64]. The deletion and the inhibition of Rac1 reduce HPV8-associated papillomatosis, while the constitutively activated form of Rac1 promotes tumorigenesis in mice [64]. This may also partly explain why we did not observe a significant contribution of IQGAP1 in the HPV16-associated HNSCC model, but further studies are needed to determine the role of Rac1/Cdc42 in PV-associated tumorigenesis, whether HPV16 and MmuPV1 are involved in the activation of Rac1 and Cdc42, and how IQGAP1 plays a role in these processes.

IQGAP1 could also impact PV-associated tumorigenesis through the immune system. T cell-mediated responses play a critical role in MmuPV1 infection and associated disease [38,71,72,73]. Interestingly, T cells lacking IQGAP1 were reported to show increased proliferation, higher T cell response, and cytokine production, indicating that IQGAP1 negatively modulates T cell activation [74,75]. Therefore, we can hypothesize that *Iqgap1^−/−^* mice are more efficient in controlling the virus via T-cell mediated mechanisms than the *Iqgap1^+/+^* mice, resulting in fewer virus-related disease. We did not observe a significant difference in viral presence at the end of 6 months post-infection based on in situ hybridization results (MmuPV1-infected *Iqgap1^+/+^* vs. MmuPV1-infected *Iqgap1^−/−^* = 90% vs. 88%, counted by the percentage of mice positive for MmuPV1 E6/E7 transcript), indicating that IQGAP1 did not affect viral clearance. However, this does not rule out the possibility that the immune response in *Iqgap1^−/−^* mice is more capable of suppressing MmuPV1-related disease progression, given that most *Iqgap1^−/−^* mice remained disease-free at the end of the study. Further investigation is needed to dissect how IQGAP1 contributes to PV-induced head and neck tumorigenesis.

## 5. Conclusions

For the first time, we reported that IQGAP1, a PI3K scaffolding protein, plays a role in PV-associated disease. With the newly developed MmuPV1 infection-based HNSCC model, more studies can now be conducted to understand the role of IQGAP1 and IQGAP1-mediated signaling at different stages of PV pathogenesis. This will provide further insight into understanding PV-mediated head and neck disease and finding new drug targets for HPV-associated cancer.

## Figures and Tables

**Table 1:** Summary of disease severity in mock- or MmuPV1-infected *Iqgap1^+/+^* and *Iqgap1^−/−^* mice.

Cohort	*n*	Normal	Dysplasia			Invasive Carcinoma
			Mild	Moderate	Severe	
Mock *Iqgap1^+/+^*	8	5	2	0	1	0
Mock *Iqgap1^−/−^*	6	4	2	0	0	0
MmuPV1 *Iqgap1^+/+^*	10	2	3	2	1	2
MmuPV1 *Iqgap1^+/+^*	9	6	1	2	0	0

## Data Availability

All data are provided in the paper and the supplementary information.

## Supplementary Materials

The following are available online at https://www.mdpi.com/article/10.3390/cancers13092276/s1, Table S1: List of antibodies used in this study, Table S2: Summary of the disease severity in *Iqgap1^+/+^*, *Iqgap1^−/−^*, *Iqgap1^+/+^K14E6E7* and *Iqgap1^−/−^K14E6E7* mice treated with 10 µg/mL 4NQO., Figure S1: qRT-PCR detecting MmuPV1 E1^E4 to confirm that MmuPV1 can infect keratinocytes, Figure S2: qPCR detecting viral presence in oral swab samples from mock- or MmuPV1-infected *Iqgap1^+/+^* and *Iqgap1^−/−^* mice at 3 weeks post-infection, Figure S3: IQGAP1 is necessary for HPV-induced PI3K signaling in vivo, Figure S4: 4NQO-treated *Iqgap1^+/+^*, *Iqgap1^−/−^*, *Iqgap1^+/+^K14E6E7*, *Iqgap1^−/−^K14E6E7* mice, Figure S5: Cancer phenotypes in 4NQO-treated *Iqgap1^+/+^*, *Iqgap1^−/−^*, *Iqgap1^+/+^K14E6E7*, *Iqgap1^−/−^K14E6E7* mice, separated by organ sites, Figure S6: IHC detecting expressions of BrdU and MCM7 in the epithelium of 4NQO-treated *Iqgap1^+/+^*, *Iqgap1^−/−^*, *Iqgap1^+/+^K14E6E7*, *Iqgap1^−/−^K14E6E7* mice, Figure S7: IF detecting expression patterns of pERK and pS6 in normal epithelium and cancers of 4NQO-treated *Iqgap1^+/+^K14E6E7* and *Iqgap1^−/−^K14E6E7* mice, Figure S8: Uncropped western blot figures.
