# Supplementary material for: Role of IQGAP1 in Papillomavirus-Associated Head and Neck Tumorigenesis
Source: Cancers (Basel). 2021 May 10;13(9):2276. doi: 10.3390/cancers13092276 (PMC8126105; doi:10.3390/cancers13092276)
Supplement: Supplementary file 1 [file cancers-13-02276-s001.zip › cancers-1168886-SI.pdf]

cancers MDPI

Supplementary Material: Role of IQGAP1 in Papillomavirus-associated Head and Neck Tumorigenesis

Tao Wei, Suyong Choi, Darya Buehler, Denis Lee, Ella Ward-Shaw, Richard A. Anderson and Paul F. Lambert

Table S1. List of antibodies used in this study.

	Name	Vendor	Catalog Number	Dilution
Westernblotting	anti-phospho-AKT	Cell Signaling	4060	1:1000
	anti-AKT	Cell Signaling	9272	1:1000
	anti-IQGAP1	Abcam	ab133490	1:1000
	anti–β-actin	Sigma		1:5000
	anti-phospho-S6	Cell Signaling	4858	1:1000
	anti-S6	Cell Signaling	2217	1:1000
	anti-HPV16 E6	GeneTex	132686	1:1000
	anti-HPV16 E7	GeneTex	133411	1:1000
	anti-tubulin	Abcam	ab7291	1:1000
Immunohistochemistry	anti-phospho-S6	Cell Signaling	4858	1:1000
	anti-phospho-ERK1/2	Cell Signaling	9101	1:100
	anti-K14	Covance	PRB-155P	1:1000
	anti-BrdU	Calbiochem	203806	1:50
	anti-MCM7	NeoMarkers	MS862	1:200

Table S2. Summary of the disease severity in *Iqgap1*$^{+/+}$, *Iqgap1*$^{-/-}$, *Iqgap1*$^{+/+}$*K14-E6E7* and *Iqgap1*$^{-/-}$*K14-E6E7* mice treated with 10 μg/mL 4NQO.

	N	Normal	Dysplasia			Invasive carcinoma			
			Mild	Moderate	Severe	Grade 1	Grade 2	Grade 3	Grade4
Iqgap1$^{+/+}$	15	10	3	0	2	0	0	0	0
Iqgap1$^{-/-}$	15	8	3	1	2	0	1	0	0
Iqgap1$^{+/+}$ *K14-E6E7*	23	1	1	0	1	9	0	6	5
Iqgap1$^{-/-}$ *K14E6E7*	23	3	0	0	3	7	2	5	3

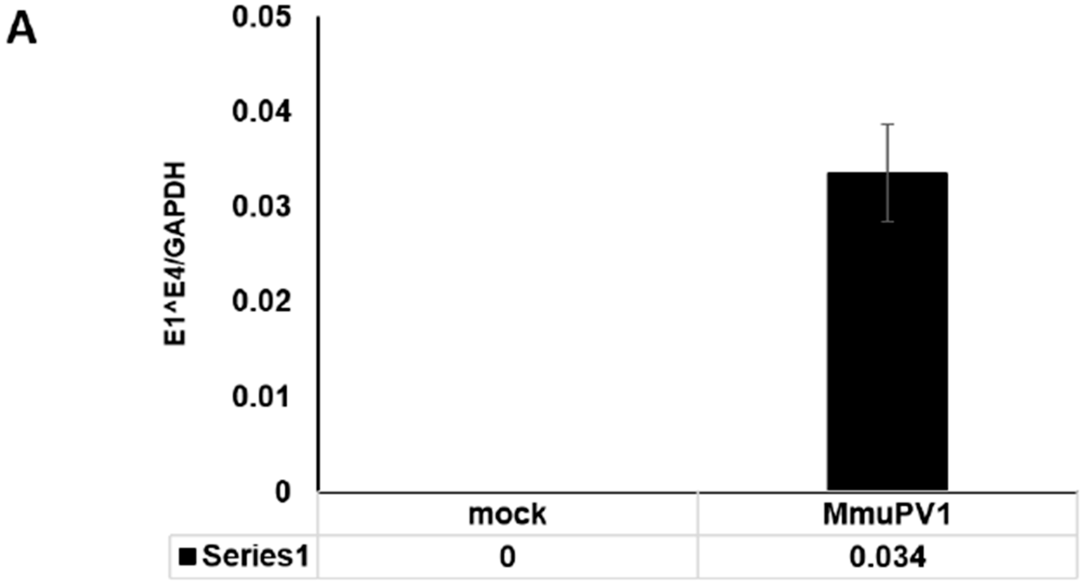

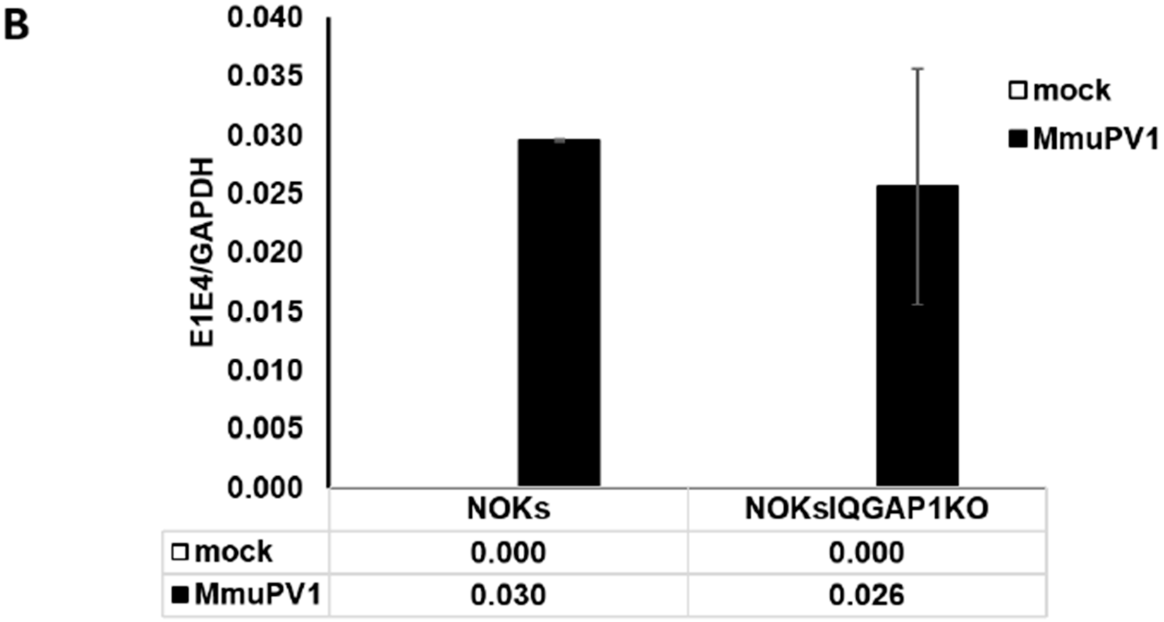

Figure S1. qRT-PCR detecting MmuPV1 E1^E4 to confirm that MmuPV1 can infect keratinocytes. (**A**) copy number of MmuPV1 E1^E4 transcript (normalized to cell host mGAPDH copy number) detected in mock- or MmuPV1-infected primary mouse keratinocytes at 48 hours post-infection. MmuPV1 E1^E4 was detected by Taqman® probe. (**B**) copy number of MmuPV1 E1^E4 transcript (normalized to cell host hGAPDH copy number) detected in mock- or MmuPV1-infected NOKs and NOKsIQGAP1KO cells at 48 hours post-infection.

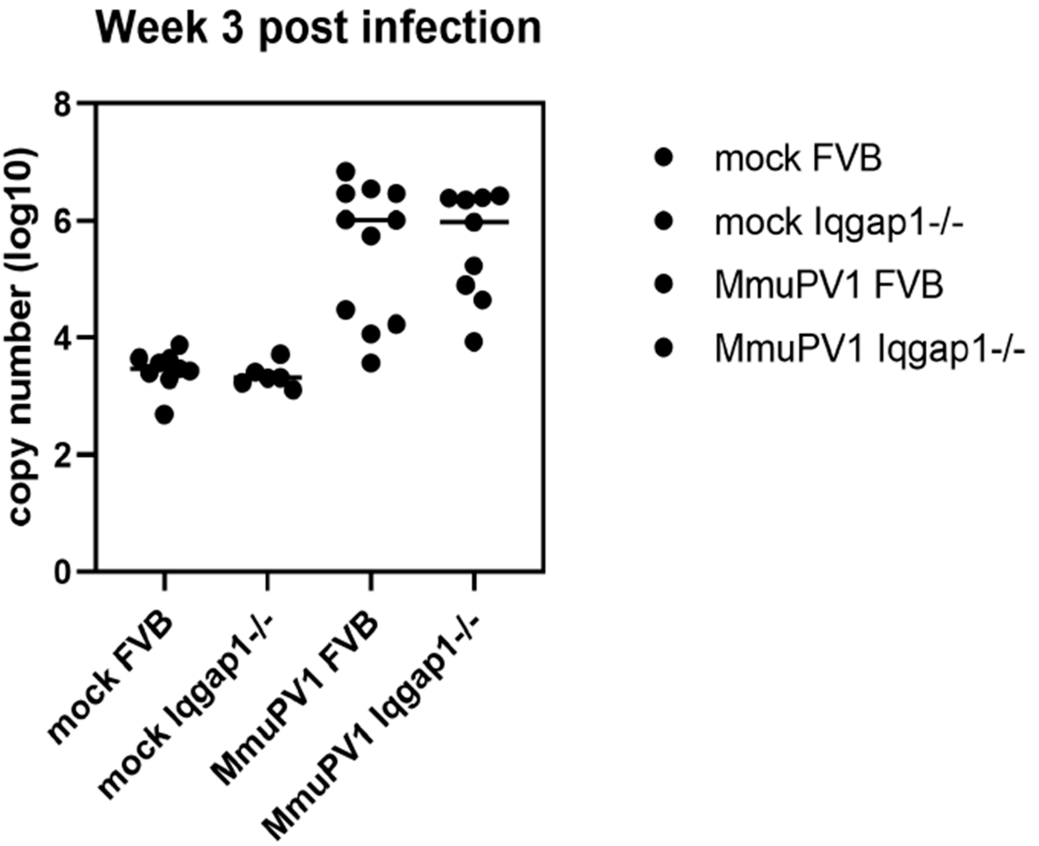

Figure S2. qPCR detecting viral presence in oral swab samples from mock- or MmuPV1-infected *Iqgap1*$^{+/+}$ and *Iqgap1*$^{-/-}$ mice at 3-week post-infection. Oral swab samples were taken from infected mice at 3-week post-infection and then proceeded for DNA extraction. qPCR was then used to detect MmuPV1 E2 gene using SYBR green method. Extracted DNA was quantified to ensure equal loading to each reaction. The result was graphed, with a line across indicating the threshold of background signal. which was determined by signals observed in negative control samples (water).

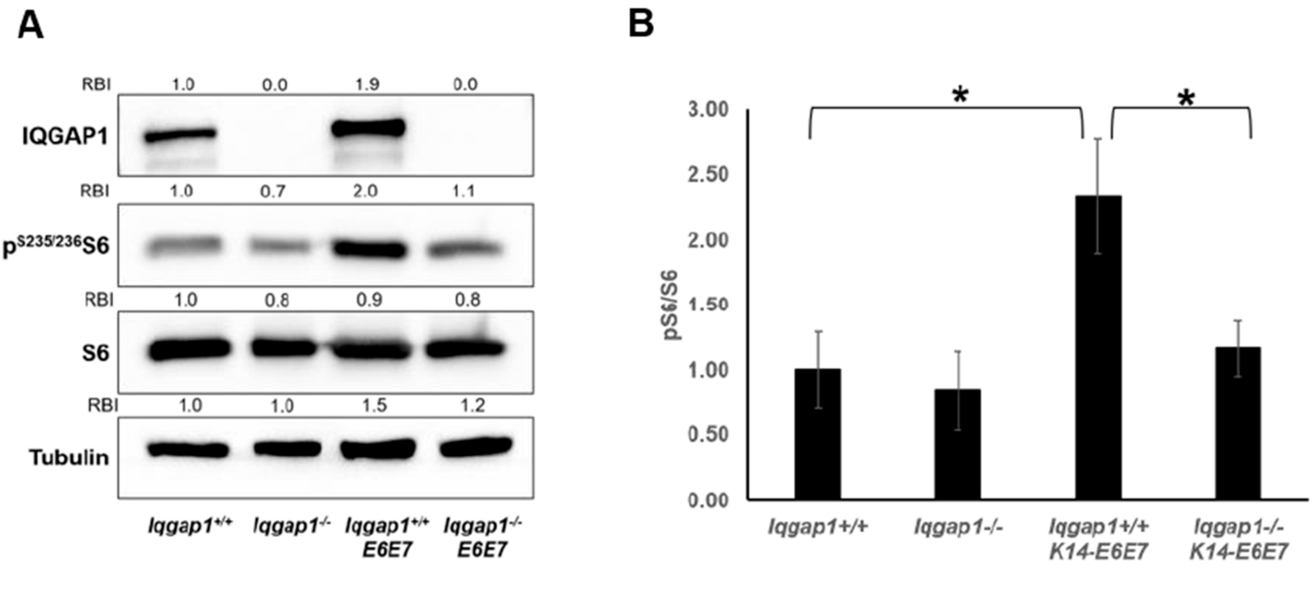

Figure S3. IQGAP1 is necessary for HPV-induced PI3K signaling *in vivo*. Back skin was collected from *Iqgap1*$^{+/+}$, *Iqgap1*$^{-/-}$, *Iqgap1*$^{+/+}$*K14E6E7*, *Iqgap1*$^{-/-}$*K14E6E7* mice and proceeded for protein lysates. (**A**) Immunoblotting to detect S6 activation. Relative band intensity (RBI) was calculated by normalizing to *Iqgap1*$^{+/+}$ lane. (**B**) quantification of (**A**) Statistics was conducted with two-sided Student T-test. Asterisk indicates statistical significance.

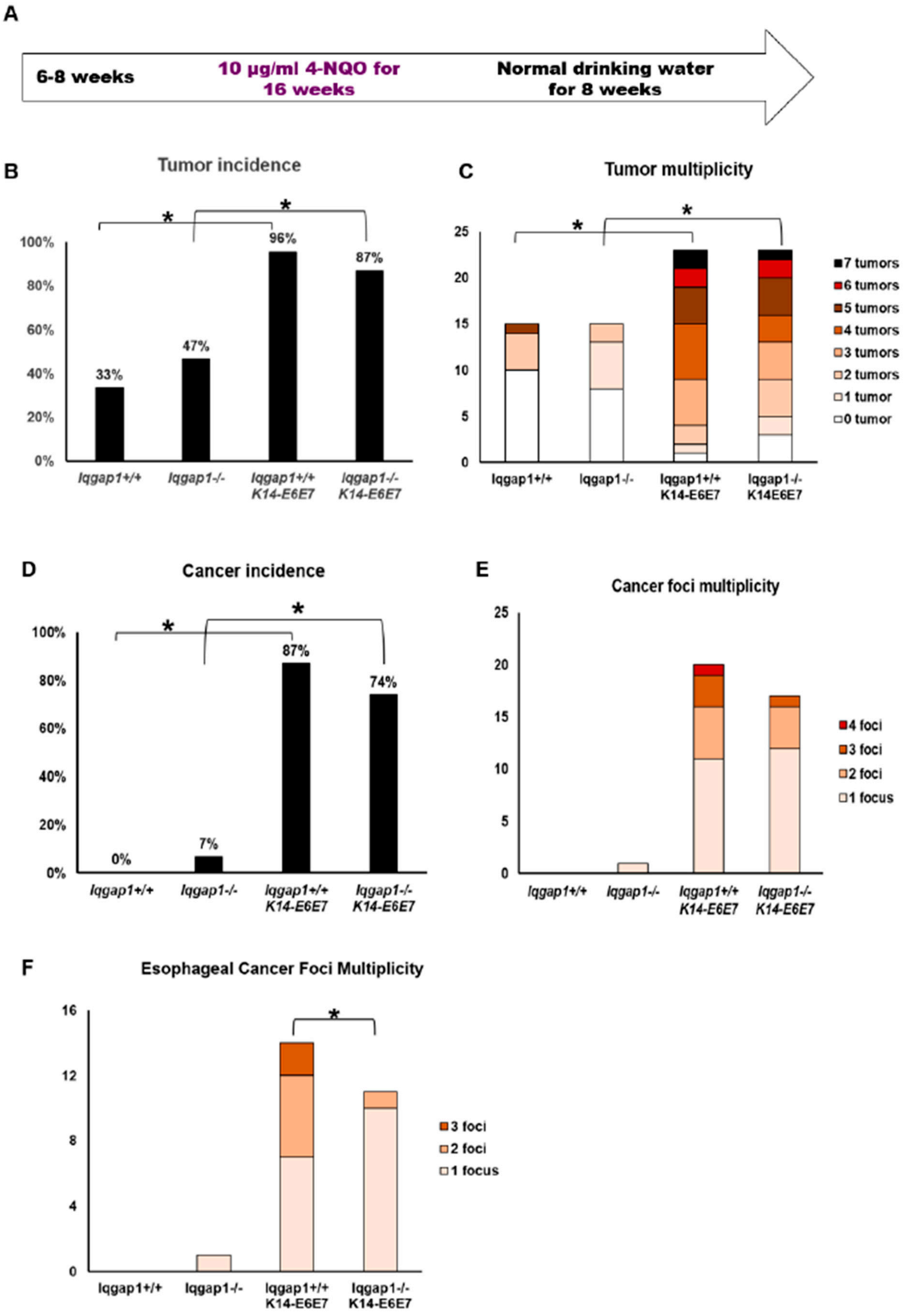

Figure S4. 4NQO-treated *Iqgap1*$^{+/+}$, *Iqgap1*$^{-/-}$, *Iqgap1*$^{+/+}$*K14E6E7*, *Iqgap1*$^{-/-}$*K14E6E7* mice. (**A**) experimental timeline. (**B**) Tumor incidence. (**C**) Tumor multiplicity. (**D**) Cancer incidence. (**E**) Cancer foci multiplicity. (**F**) esophageal cancer foci multiplicity. Asterisk indicates statistical significance.

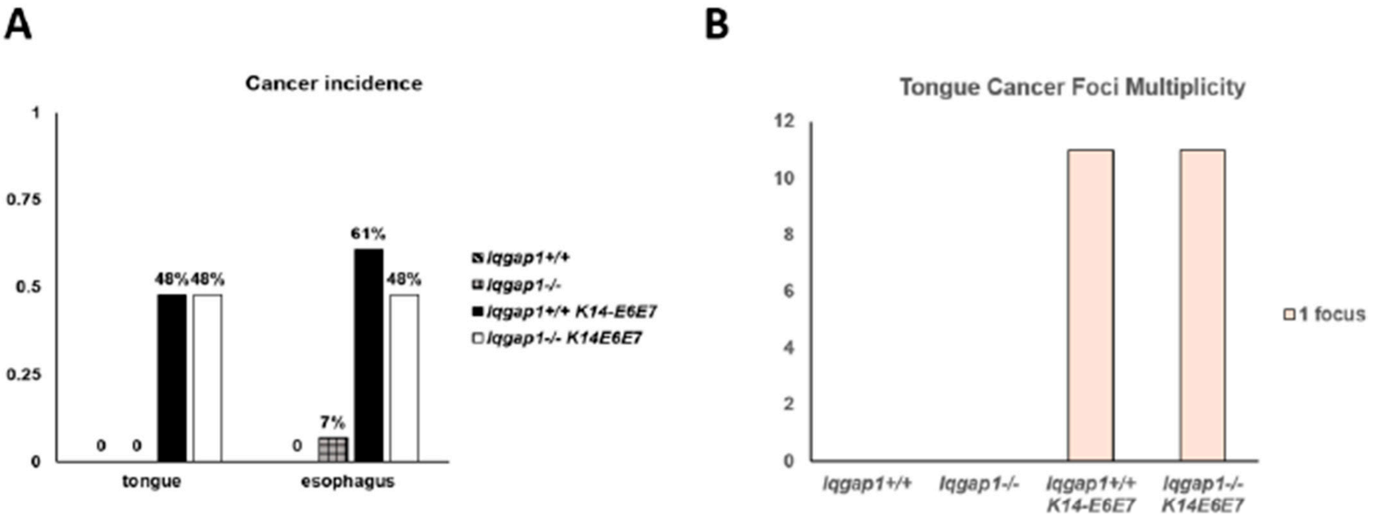

C Disease Severity summary of tongue tumors

	N	Normal	Dysplasia			Invasive carcinoma			
			Mild	Moderate	Severe	Grade 1	Grade 2	Grade 3	Grade4
Iqgap1 $^{+/+}$	15	11	3	1	0	0	0	0	0
Iqgap1 $^{-/-}$	15	14	0	1	0	0	0	0	0
Iqgap1 $^{+/+}$ *K14-E6E7*	23	5	3	0	4	9	0	2	0
Iqgap1 $^{-/-}$ *K14E6E7*	23	5	0	2	5	6	2	2	1

D Disease Severity summary of esophageal tumors

	N	Normal	Dysplasia			Invasive carcinoma			
			Mild	Moderate	Severe	Grade 1	Grade 2	Grade 3	Grade4
Iqgap1 $^{+/+}$	15	10	3	0	2	0	0	0	0
Iqgap1 $^{-/-}$	15	9	3	0	2	0	1	0	0
Iqgap1 $^{+/+}$ *K14-E6E7*	23	2	1	0	6	5	0	4	5
Iqgap1 $^{-/-}$ *K14E6E7*	23	5	1	0	6	4	1	4	2

Figure S5. Cancer phenotypes in 4NQO-treated *Iqgap1* $^{+/+}$, *Iqgap1* $^{-/-}$, *Iqgap1* $^{+/+}$*K14E6E7*, *Iqgap1* $^{-/-}$*K14E6E7* mice, separated by organ sites. (**A**) Cancer incidence. Statistical analysis was conducted with Fisher's exact test: *Iqgap1* $^{+/+}$*K14E6E7* vs. *Iqgap1* $^{-/-}$ *K14E6E7*, tongue: $p = 1$; esophagus: $p = 0.46$. (**B**) Tongue cancer foci multiplicity. (**C**) Disease severity of tongue cancer. Statistical analysis was conducted with Wilcoxon rank sum test: *Iqgap1* $^{+/+}$*K14E6E7* vs. *Iqgap1* $^{-/-}$*K14E6E7*= 3.7 vs. 4.1, $p = 0.63$. (**D**) Disease severity of esophageal cancer. Statistical analysis was conducted with Wilcoxon rank sum test: *Iqgap1* $^{+/+}$*K14E6E7* vs. *Iqgap1* $^{-/-}$*K14E6E7* = 5.2 vs. 4.2, p=0.21.

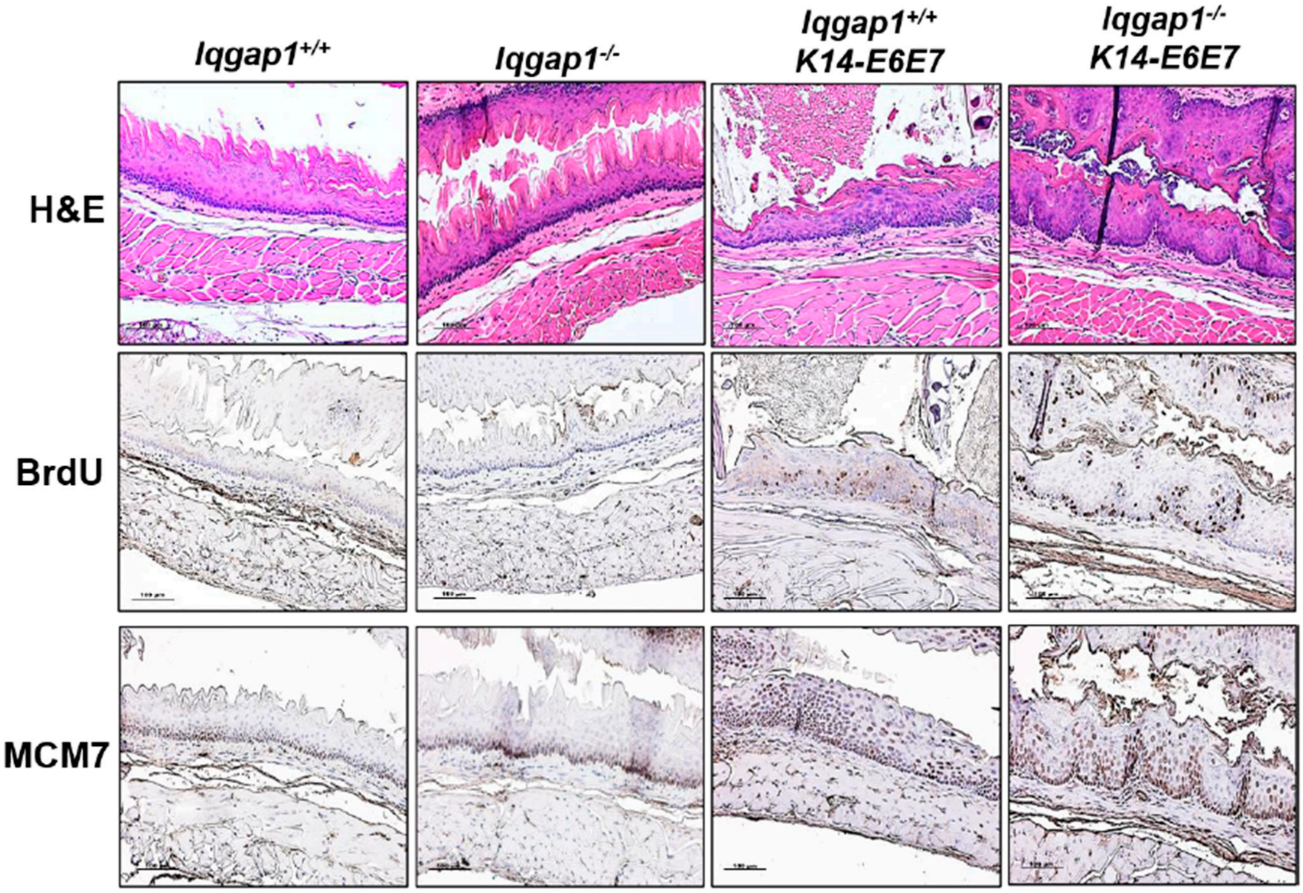

Figure S6. IHC detecting expressions of BrdU and MCM7 in the epithelium of 4NQO-treated *Iqgap1*$^{+/+}$, *Iqgap1*$^{-/-}$, *Iqgap1*$^{+/+}$*K14E6E7*, *Iqgap1*$^{-/-}$*K14E6E7* mice.

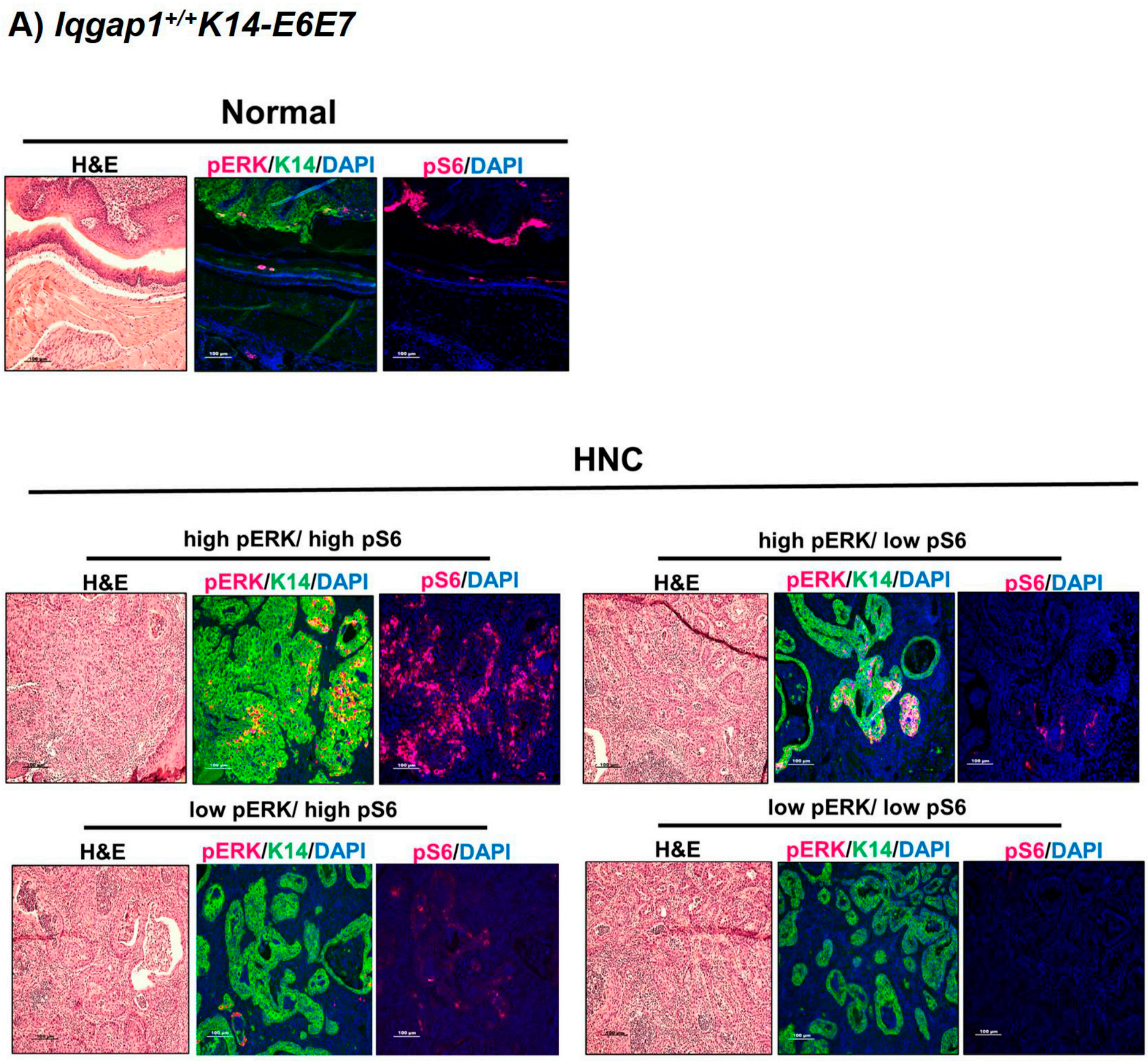
A) Iqgap1+/+K14-E6E7
Normal
H&E
pERK/K14/DAPI
pS6/DAPI
HNC
high pERK/ high pS6
high pERK/ low pS6
low pERK/ high pS6
low pERK/ low pS6
100 µm

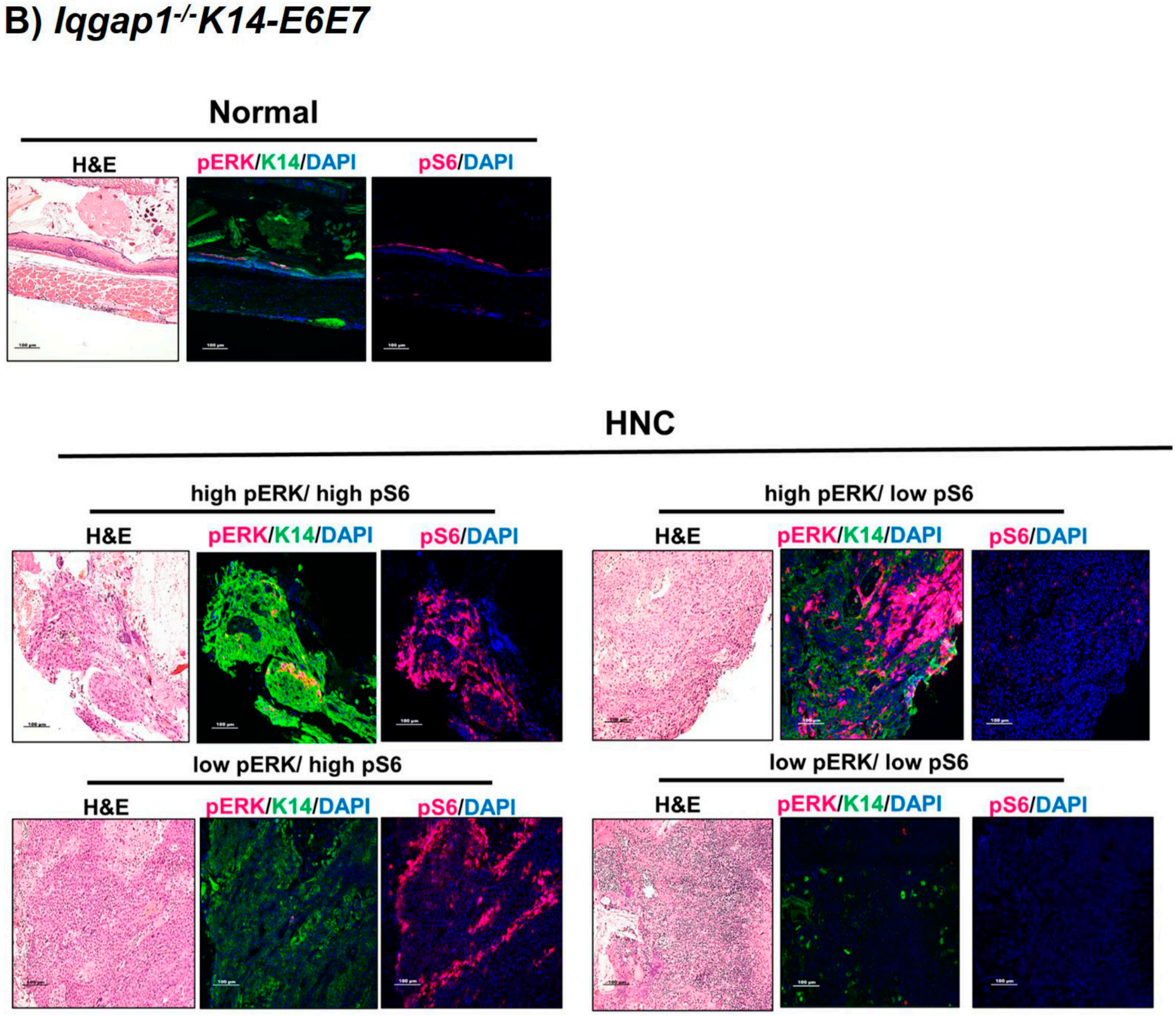

Figure S7. IF detecting expression patterns of pERK and pS6 in normal epithelium and cancers of 4NQO-treated Iqgap1+/+K14E6E7 (panel **A**) and Iqgap1-/-K14E6E7 (panel **B**) mice.

Original blot images of Figure 1A

Piece A: IQGAP1

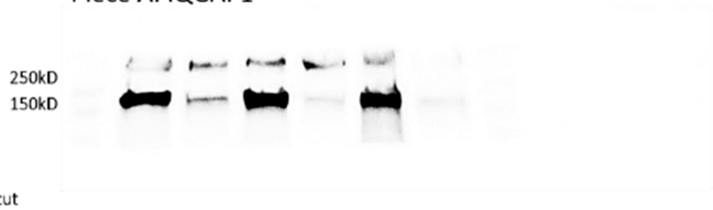

A&B were cut between 75 and 100kD markers

Piece B: actin

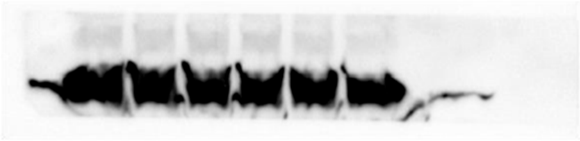

B&C were cut along the lower edge of 37kD marker

Piece C: HPV16 E6

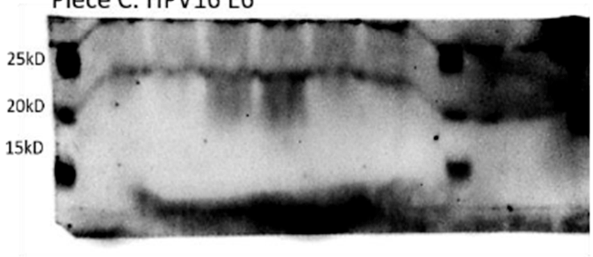

Same blot cut into 3 pieces to probe separately

Piece C: HPV16 E7

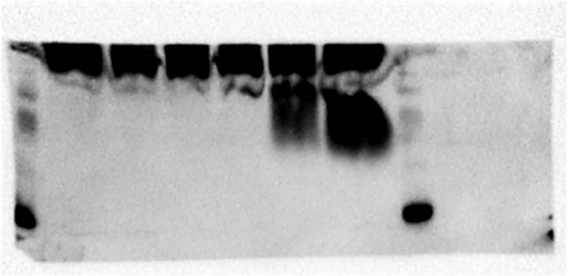

Piece C was stripped& reprobed for E7

Original blot images of Figure 1B

Piece A: IQGAP1

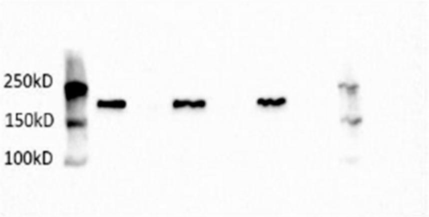

A&B were cut along the lower edge of 100kD marker

Piece B: pAKT

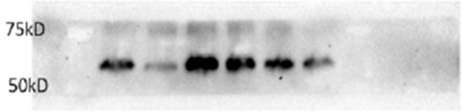

B&C were cut along the lower edge of 50kD marker

Piece C: actin

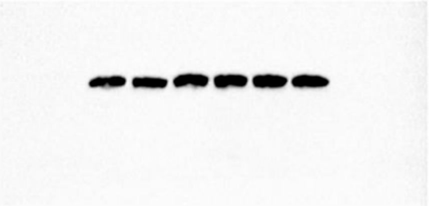

Piece B: AKT

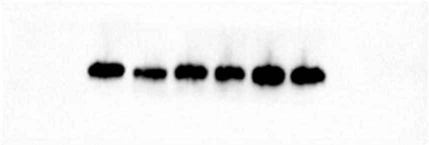

Piece B was stripped& reprobed for AKT

Same blot was cut into 3 pieces to probe separately

Original blot images of Figure 2A

* Rectangled regions were the portion selected for the main figure

Piece A: Not shown- not part of this manuscript

A&B were cut between 75& 100kD marker

Piece B: actin

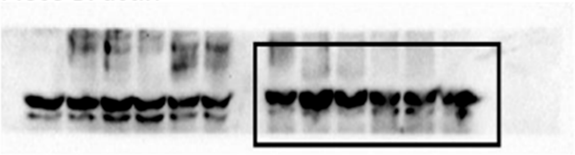

B&C were cut along the lower edge of 37kD marker

Piece C: pS6

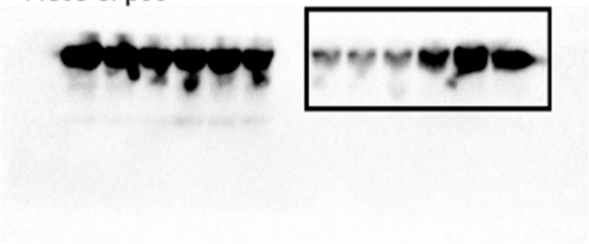

Same blot was cut into 3 pieces to probe separately

Piece C: S6

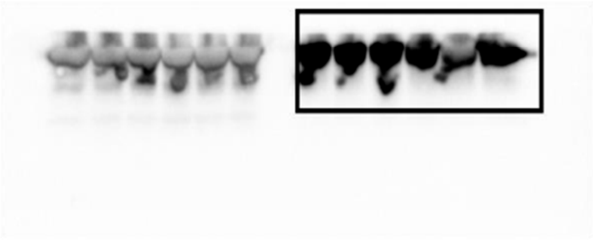

Piece C was stripped& reprobed for S6

Original blot images of Figure 2B

Piece A: IQGAP1

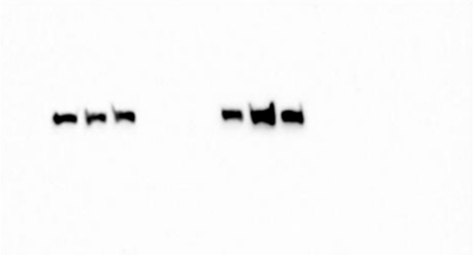

A&B were cut between 75& 100kD marker

Piece B: pAKT

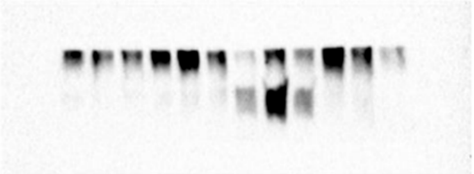

B&C were cut along the lower edge of 50kD marker

Piece C: actin

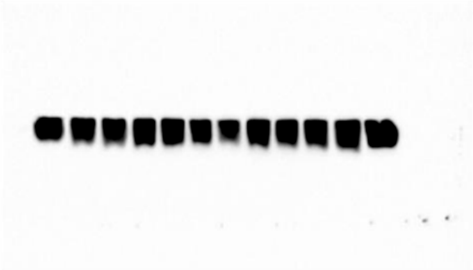

Same blot was cut into 3 pieces to probe separately

Piece B: AKT

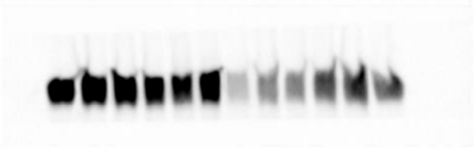

Piece B was stripped& reprobed for AKT

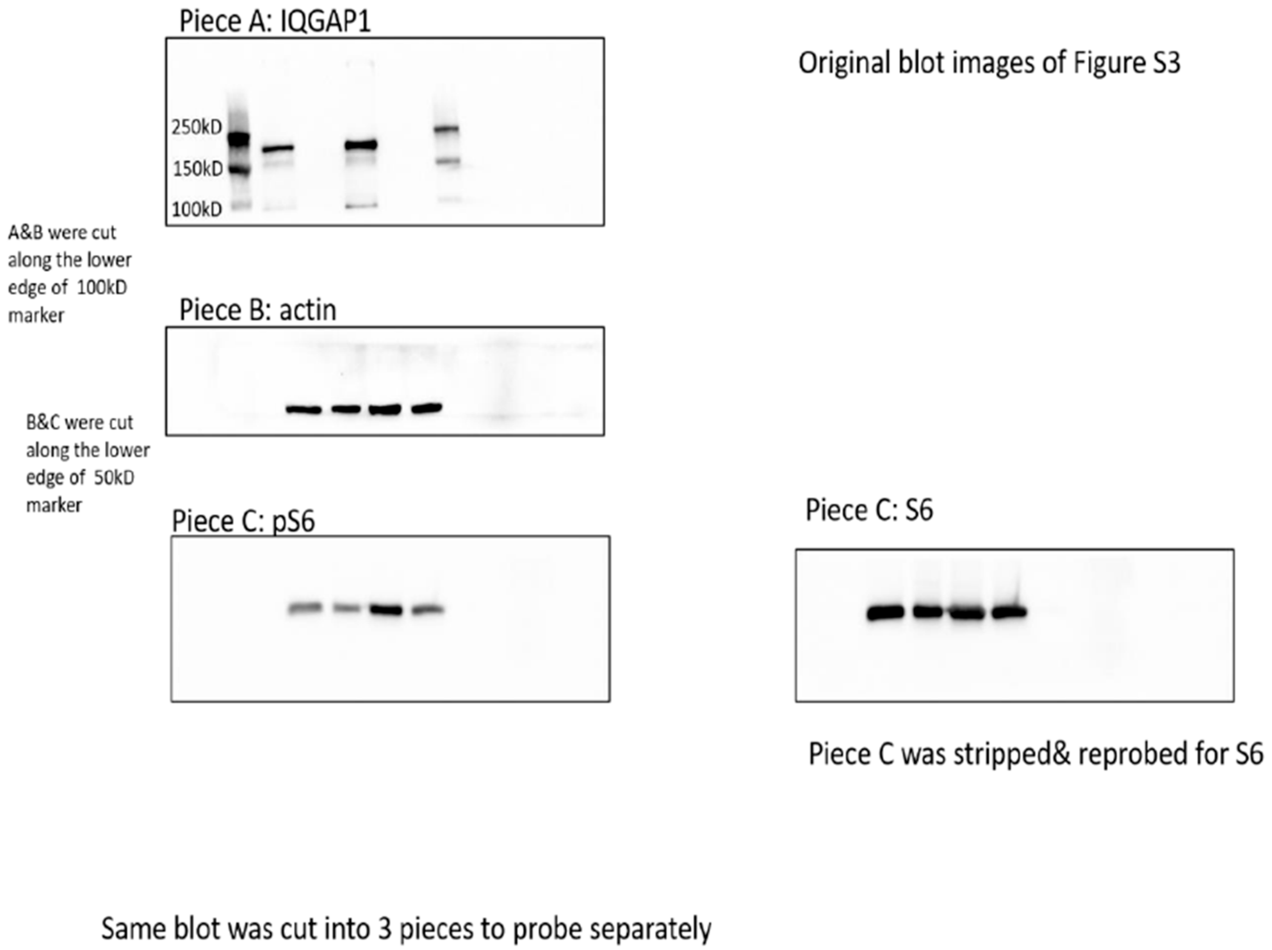

Figure S8. Uncropped Western blot figures.
